# Effects of adding a diet intervention to exercise on hip osteoarthritis pain: protocol for the ECHO randomized controlled trial

**DOI:** 10.1186/s12891-022-05128-9

**Published:** 2022-03-05

**Authors:** Michelle Hall, Rana S. Hinman, Gabrielle Knox, Libby Spiers, Priya Sumithran, Nicholas J. Murphy, Fiona McManus, Karen E. Lamb, Flavia Cicuittini, David J. Hunter, Stephen P. Messier, Kim L. Bennell

**Affiliations:** 1 Centre for Health, Exercise and Sports Medicine, Department of Physiotherapy, University of Melbourne, Melbourne, Victoria Australia; 2 Department of Medicine (St Vincent’s), University of Melbourne, Melbourne, Australia; 3 Department of Endocrinology, Austin Health, Melbourne, Australia; 4 Institute of Bone and Joint Research, Kolling Institute, University of Sydney, Sydney, Australia; 5 Department of Orthopaedic Surgery, John Hunter Hospital, Newcastle, Australia; 6 Centre for Epidemiology and Biostatistics, Melbourne School of Population and Global Health, University of Melbourne, Melbourne, Australia; 7 MISCH (Methods and Implementation Support for Clinical Health research platform), Faculty of Medicine, Dentistry and Health Sciences, The University of Melbourne, Melbourne, Victoria Australia; 8 School of Public Health and Preventative Medicine, Monash University, Melbourne, Australia; 9 J.B. Snow Biomechanics Laboratory, Department of Health Exercise Science, Wake Forest University, Winston-Salem, North Carolina USA; 10 Section on Gerontology and Geriatric Medicine, Wake Forest School of Medicine, Winston-Salem, North Carolina USA

**Keywords:** Osteoarthritis, Exercise, Physical activity, Weight management, Ketogenic diet, Hip, Pain, Obesity

## Abstract

**Background:**

Hip osteoarthritis (OA) is a leading cause of musculoskeletal pain. Exercise is a core recommended treatment. Despite some clinical guidelines also recommending weight loss for hip OA, there is no evidence from randomised controlled trials (RCT) to substantiate these recommendations. This superiority, 2-group, parallel RCT will compare a combined diet and exercise program to an exercise only program, over 6 months.

**Methods:**

One hundred people with symptomatic and radiographic hip OA will be recruited from the community. Following baseline assessment, participants will be randomly allocated to either, i) diet and exercise or; ii) exercise only. Participants in the diet and exercise group will have six consultations with a dietitian and five consultations with a physiotherapist via videoconferencing over 6 months. The exercise only group will have five consultations with a physiotherapist via videoconferencing over 6 months. The exercise program for both groups will include prescription of strengthening exercise and a physical activity plan, advice about OA management and additional educational resources. The diet intervention includes prescription of a ketogenic very low-calorie diet with meal replacements and educational resources to support weight loss and healthy eating. Primary outcome is self-reported hip pain via an 11-point numeric rating scale (0 = ‘no pain’ and 10 = ‘worst pain possible’) at 6 months. Secondary outcomes include self-reported body weight (at 0, 6 and 12 months) and body mass index (at 0, 6 and 12 months), visceral fat (measured using dual energy x-ray absorptiometry at 0 and 6 months), pain, physical function, quality of life (all measured using subscales of the Hip Osteoarthritis Outcome Scale at 0, 6 and 12 months), and change in pain and physical activity (measured using 7-point global rating of change Likert scale at 6 and 12 months). Additional measures include adherence, adverse events and cost-effectiveness.

**Discussion:**

This study will determine whether a diet intervention in addition to exercise provides greater hip pain-relief, compared to exercise alone. Findings will assist clinicians in providing evidence-based advice regarding the effect of a dietary intervention on hip OA pain.

**Trial registration:**

ClinicalTrials.gov . Identifier: NCT04825483. Registered 31st March 2021.

**Supplementary Information:**

The online version contains supplementary material available at 10.1186/s12891-022-05128-9.

## Background

Hip OA is a major public health problem and symptomatic hip OA affects one in four adults over their lifetime [1]. There is no cure for OA and joint replacement is reserved for end-stage disease. Of concern is the life-time risk of hip replacement for hip OA is as high as 1 in 7 for women and 1 in 10 for men [2]. In 2018, 77% of the total hip joint replacements performed in Australia were for people with hip OA with comorbid overweight/obesity [3]. Effective treatments to reduce hip OA pain are urgently needed.

Clinical guidelines [4–8] recommend exercise as the core treatment for hip OA symptoms. Systematic reviews and meta-analyses of clinical trials evaluating land-based exercise report small-to-moderate benefits for hip OA pain [9, 10]. Muscle weakness is common in hip OA [11] and the majority of evidence for exercise is strength-related [9]. In addition to strengthening exercise, regular physical activity is important as people with OA are at increased of risk of death due to cardiovascular disease [12]. Indeed, many people with hip OA do not meet physical activity recommendations [13]. Exercise may indeed delay hip joint replacement for some people with hip OA [14]. However, up to 85% of people with hip OA have overweight or obesity [15] and exercise therapy in isolation may be insufficient to reduce pain.

Recommendations for weight loss to manage hip OA symptoms are inconsistent across clinical guidelines, due to the absence of clinical trials in hip OA evaluating weight loss. Clinical guidelines [4, 6–8] including the 2019 American College of Rheumatology guidelines [8], recommend weight loss for hip OA based on evidence from clinical trials in knee OA. However, the 2019 Osteoarthritis Research Society International guidelines for hip OA management do not recommend weight loss for hip OA [5] due to the absence of clinical trials in hip OA evaluating weight loss. A reduction in body weight by at least 5% is recommended for improvement in clinical and mechanistic outcomes, however this is based on evidence in people with knee OA [8]. Irrefutable health benefits are associated with weight loss for those with overweight or obesity [16]. However, our systematic review and meta-analysis of clinical trials found that a combination of diet and exercise reduced knee OA pain compared to control, but not for diet only interventions compared to control [17]. Notably, the control was considered either an active (non-diet treatment) or no treatment (including placebo or waiting list) [17]. It remains uncertain whether weight loss in addition to exercise and regular physical activity is more benefical for hip OA pain compared to exercise alone.

Among the myriad of weight loss strategies, the ketogenic very low calorie diet (VLCD) leads to greater short-term weight loss compared to low-fat diets [18] and low calorie diets [19]. This program is typically characterised by an intensive weight loss phase followed by a transition and maintenance phase. A systematic review and meta-analysis of ketogenic VLCDs found an average 14% reduction in body weight loss in studies with a ketogenic phase up to 12 weeks with loss in body weight stable up to 2 years follow-up [19]. Additionally, a VLCD was associated with improvements in factors related to cardiovascular disease such as blood pressure, total cholesterol and triglycerides. Importantly, in combination with exercise, using nutritionally complete meal replacement products is considered safe in adults aged up to 80 years or older [20]. A VLCD is perceived as acceptable to people with knee OA [21], and our pilot study in hip OA (*n* = 18) demonstrated feasibility of a ketogenic VLCD and exercise intervention delivered remotely [22]. We observed an average of 10% [95% CI 8 to 12%] reduction in body weight at 3 months and no adverse events related to the diet program were reported [22].

The primary aim of this randomised controlled trial (RCT) is to determine if a 6-month diet and exercise program results in significantly greater improvements in hip pain compared to an exercise only program at 6 months. We hypothesise that diet and exercise will reduce pain more than exercise alone.

The secondary aims of this study are to test the hypothesis that: i) a diet and exercise program will improve hip pain more than exercise alone at 12-months; ii) a diet and exercise program will improve other clinical outcomes (physical function, body composition, quality of life; physical activity) at 6 months and 12 months.

## Methods

### Design and management

The ECHO trial is a superiority, 2-group, parallel RCT. The trial will be based in Melbourne, Australia. The University of Melbourne Human Research Ethics Committee approved the study (HREC 201516) and the trial was prospectively registered with ClinicalTrials.gov (NCT04825483). The trial is sponsored by the University of Melbourne. The trial was designed according to the Consolidated Standards of Reporting Trials statement [23] and is reported according to the Standard Protocol Items: Recommendations for Interventional Trials statement [24]. The current study protocol (version 2) is available online as Additional File 1 and any protocol amendments will be detailed in the trial registration following ethics approval. All participants will provide written informed consent. Due to the expected low risk of harm based on our pilot study [22], we have not planned a data safety monitoring committee. There is also no planned interim analysis or stopping guidelines.

### Participants

We will recruit 100 participants with hip OA from metropolitan Melbourne and Sydney through advertisement, print/radio/social media and our research volunteer database. Hip OA will be classified according to the American College of Rheumatology classification criteria (pain in the groin or hip region on most days of the past month and femoral or acetabular osteophytes and joint space narrowing (superior, axial and/or medial) on x-ray) [25]. Participants will be included if they:

i) are aged 50 years or older;
ii) report a history of hip pain ≥3 months;
iii) report an average pain score of at least 4 on an 11-point numeric rating scale (anchored at 0 = no pain, 10 = worst pain imaginable) over the previous week;
iv) report pain in the groin or hip region on most days of the past month [25];
v) have femoral or acetabular osteophytes and joint space narrowing (superior, axial and/or medial) on x-ray) [25];
vi) have access to a device with internet connection;
vii) have a BMI > 27 kg/m^2^; based on the Royal Australian College of General Practitioners guideline for obesity management, which indicates usage of a very low energy diet to induce rapid weight loss for those with BMI > 30 kg/m^2^ or those with BMI > 27 kg/m^2^ who also suffer comorbidities such as OA [26];
viii) are willing and able to give informed consent and participate fully in the interventions and assessment procedures;
ix) have the ability to weigh themselves (e.g. access to scales);
x) pass the Exercise and Sports Science Australia stage 1 adult pre-exercise screening system [27] or obtain general practitioner clearance for participation in the study.

Participants will be excluded if they:

i) weigh > 150 kg (due to the added complexities of additional nutritional requirements for individuals above this weight);
ii) are unable to speak and read English;
iii) are on a waiting list for/planning back/lower limb surgery or bariatric surgery in the next 12 months;
iv) have had previous arthroplasty on affected hip;
v) have had recent hip surgery on affected hip (past 6 months);
vi) have self-reported inflammatory arthritis (e.g. rheumatoid arthritis);
vii) lost > 2 kg weight over the previous 3 months (to ensure that participants were not already engaging in weight change behaviour)
viii) are already actively trying to lose weight by any of the following mechanisms:

- using meal replacements for weight loss;
- being a member of a commercial weight loss program (e.g. Weight Watchers);
- receiving support from any health care professional for weight loss (i.e. participating in ongoing consultations about weight loss with a health care professional);
- using any drugs prescribed to aid in weight loss;
- using structured meal programs for weight loss such as ‘Lite n’ Easy’;

ix) are unable to undertake a ketogenic VLCD without close medical supervision including self-reported:

- diagnosis of Type 1 diabetes;
- Type 2 diabetes requiring insulin or other medication apart from metformin;
- warfarin use;
- stroke or cardiac event in previous 6 months;
- unstable cardiovascular condition;
- fluid intake restriction;
- renal (kidney) problems (unless clearance is obtained from GP, including GP confirmation that estimated glomerular filtration rate > 30 mL/min/1.73m^2^);

xxiv) have any neurological condition affecting lower limbs;
xxv) are pregnant or planning pregnancy;
xxvi) have vegan dietary requirements (due to the complexity of delivering a nutritionally complete diet within the ketogenic diet regime).

### Procedure

**Fig. 1:**
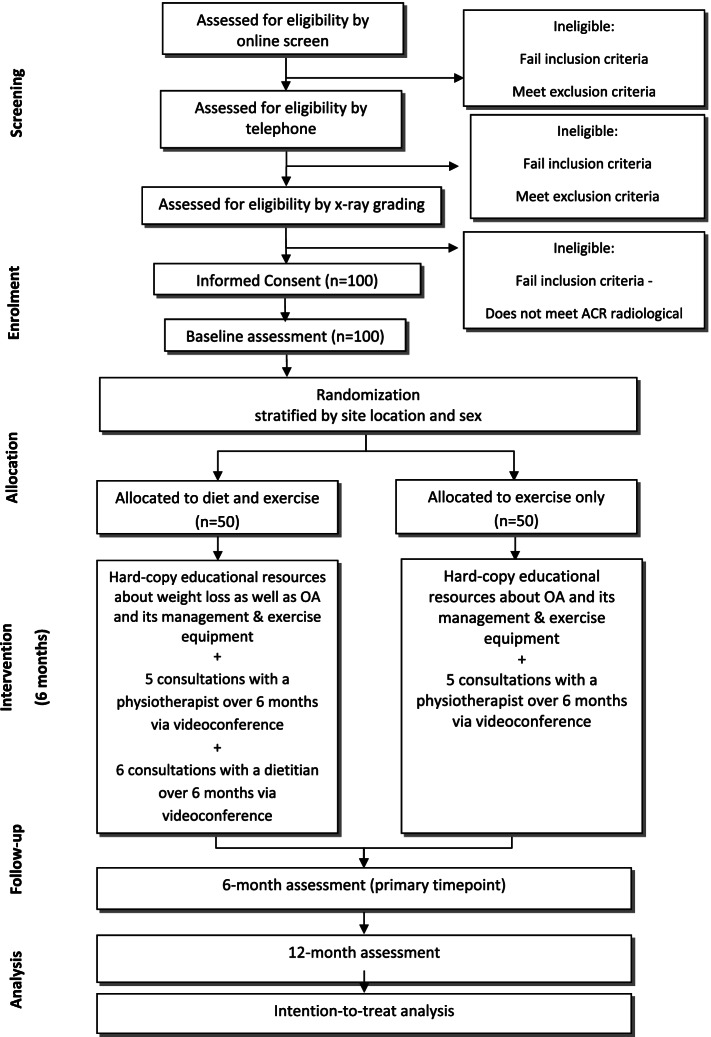
Flow diagram of study phases

The trial phases are outlined in Fig. 1. Volunteers will initially be screened by an online form, then over the phone by research staff at The University of Melbourne. Volunteers may also call a telephone number to proceed directly to phone screening if preferred to completing the online screening. Additional clearance to participate will be sought from a general practitioner for anyone who does not pass the pre-exercise screening questions and/or indicates that they suffer with renal dysfunction. During the telephone screening process the Trial Coordinator will provide a verbal description of the project to ensure that participants fully understand the trial procedures. If participants pass the eligibility criteria assessed during the telephone screening process, they will receive the Plain Language Statement (PLS) and Consent Form (see Additional File 2) in the post or by email. After reading the PLS, and if they give their consent to participate, consent will be obtained online using REDCap or they will sign a paper-based consent form and return it via a reply-paid envelope in the post or by scanning and emailing the document to the Trial Coordinator. Screening information and study consent forms will be stored within a secure data collection platform (Qualtrics or REDCap) and accessible only by password to the researchers. Potentially eligible participants will undergo an antero-posterior supine hip x-ray. Participants who have undergone an antero-posterior supine hip x-ray in the prior 12 months and can provide the images and reports to the research staff for screening will not undergo new x-rays. It is deemed unnessary to expose these partcipants to additional radiation. For participants with bilaterally eligible hips, the most symptomatic hip will be deemed the study hip with respect to outcome measurement.

### Data collection and management

Body composition scans and x-rays will be collected at clinics in Melbourne and Sydney and will be stored on a secure password-protected server. Other participant-reported or clinician reported outcomes will be collected electronically via computer or paper (if preferred) at all times. If questionnaires are not completed or returned, the participant will be contacted by email and/or phone to prompt completion and return, or as a last resort, to obtain primary outcome data over the phone. Paper questionnaires will be stored in locked filing cabinets, and the data will be manually entered into REDCap by the Trial Coordinator. Electronic copies will be stored in the REDCap website, accessible only to the researchers by password protection.

### Randomisation and allocation concealment

Eligible participants will be randomised to receive either i) diet and exercise or ii) exercise. An independent biostatistician has prepared the randomisation schedule (random permuted blocks) stratified by site (Melbourne or Sydney) and sex (male or female). The schedule will be stored on a password-protected website (REDCap) at The University of Melbourne maintained by a researcher not involved in either participant recruitment or administration of outcome measures. Each participant will receive a unique study ID code, and this will be documented in the participant’s record/database in addition to all study documents. Group allocation will be revealed after completion of baseline outcomes.

Although participants will not be informed about the study hypothesis until the study is completed, the participants will be unblinded. Participants will be informed that we are comparing the two interventions (described below) to determine which intervention achieves the most benefits. The primary outcomes and some of the secondary outcomes are participant-reported, so participants are also the assessors. Physiotherapists and dietitians will not be blinded to group allocation or study hypothesis. Staff conducting assessments for some of the secondary outcomes (e.g. body composition) will be blinded. Statistical analyses will be performed blinded.

### Interventions

The interventions are based on a diet and exercise program developed by the researchers for people with knee OA [28], with input from various stakeholders, and are reported according to TIDIeR recommendations [29]. Clinical consultations are informed by the Behaviour Change Wheel [30] and based on research into exercise [28, 31] and weight management [22] for hip and knee OA. Prior to the first consultation with the physiotherapist and dietitian, participants will complete a pre-consultation survey asking about their main problems and goals, brief history of their hip symptoms and other health issues and previous weight management if they are allocated to the weight loss and exercise group.

### Exercise

In addition to receiving hardcopy educational resources (Table 1) via post, participants in both groups will have 5 videoconference individual consultations with a physiotherapist over 6 months. The timing of these consultations is recommended in weeks 1, 3, 9, 15 and 21, but will be negotiated between the participant and their physiotherapist. The first consultation will last for approximately 45 min, with the remaining consultations to last approximately 30 min. Participants will receive four exercise resistance bands of varying resistance (yellow, green, red and blue), an ankle cuff-weight (1.0 to 5.0 kg), a booklet of exercise instructions (along with progressions) and an exercise logbook to record their program and what exercises they complete. The exercise logbooks are used as motivational tool for participants and for their physiotherapist to assess their progress through the exercise program. Physiotherapists will i) support participants to engage in a structured and progressive muscle strengthening program; ii) support participants to engage in a physical activity plan to increase incidental and general physical activity; iii) identify barriers to exercise and approaches to overcome these; iv) advise how to self-monitor and manage flare-ups, and v) review, progress and modify program as appropriate.

**Table 1:** Summary of resources provided to participants by group allocation

Resource	Description	Diet and Exercise group	Exercise only group
Consultations with a physiotherapist	5-video consultations over 6-months. Provides structured exercise and physical activity plan and behaviour change support	✓	✓
Consultations with a dietitian	6-video consultations over 6-months. Supports participant to undertake ketogenic VLCD, including behaviour change techniques	✓	
Exercise bands	4 exercise resistance bands (yellow, red, green, blue) for strengthening exercises	✓	✓
Exercise weights	Ankle cuff weight	✓	✓
Plastic portion plate	Assist with portion sizes	✓	
Optifast® meal replacements	Up to 6-months’ supply of meal replacement for the ketogenic VLCD	✓	
Educational video about the VLCD	Short video about the ketogenic VLCD featuring endocrinologists and dietitian experts, and a person with OA	✓	
**Booklets**			
Preparing for your consultations	Details about consultations, instruction on how to use Zoom videoconferencing	✓	✓
Osteoarthritis information	Information about osteoarthritis, typical management options, weight loss, pain coping skills and sleep	✓	✓
Exercise booklet	Strengthening exercise instructions and photographs	✓	✓
Hip exercise plan and log-book	Templates to record details of management plans and complete exercises	✓	✓
Weight management ‘how to’ guide	Describes the ketogenic VLCD and provides information about healthy food choices and portion sizes	✓	
Weight management behavioural support activities	Workbook that contains information and templates to track weight, a food diary, tips to find a support person, identify food triggers, plans for ‘at risk’ scenarios, overcoming barriers changing thought patterns, and monitoring hunger levels	✓	
Recipe book	Suitable recipes for ketogenic VLCD	✓	
Food list pocket guide	Low carbohydrate ingredients to consume when on the ketogenic VLCD	✓	

*VLCD* Very low-calorie diet, *OA* Osteoarthritis

The strengthening exercise program is to be completed 3 times per week and includes exercises that target the hip extensors, hip flexors, hip abductors, hip adductors and knee extensors. Physiotherapists will prescribe 4–6 exercises (Table 2), at an intensity where participants feel that is “hard to very hard” to complete a full set of each exercise (equivalent to 5–7 on the Borg CR 10 Rating of Perceived Exertion) [32]. The volume (i.e. set and repetitions) will be negotiated between the physiotherapist and participant. During the consultations, physiotherapists will i) review the logbook to assess adherence and progress; ii) modify the strengthening program as needed; iii) provide feedback on technique and; iv) discuss any flare-ups or adverse events. For participants in the weight loss and exercise group, physiotherapists will also prescribe arm strengthening exercises to minimise loss of muscle mass, which can occur with weight loss [33]. For the physical activity plan, physiotherapists will encourage the participant to increase their general and incidental levels of physical/aerobic activity based on their individual need and goals, as well as their current level of activity. Participants will be encouraged to keep going with the exercise program after consultations with the physiotherapists finish.

**Table 2:** Home based strengthening exercise protocol

Minimum of 4 and maximum of 6 exercises, with progression as appropriate:			
1 Hip extensor exercise			
1 Hip flexor exercise			
1 Hip abductor exercise			
1 Functional/knee extensor exercise			
Once a participant is able to do more than the minimum of 4 exercises, an exercise will be added from the hip adductor group or an additional exercise from the above groups.			
**Hip Extensors**			
Leg lift	Non-weight bearing	Face-down leg lift	**Instruction:** Lie on stomach with hands folded under your chin. Bend the knee on the same side as your study hip and push heel towards ceiling, lifting leg off the bed and squeezing buttocks the whole time. Hold for 1–2 s, then lower leg.
	Non-weight bearing	4-point-kneel leg lift	**Instruction:** On all fours, slowly kick the study leg backward, squeezing buttocks and maintain 90degree bend in the knee. Bring leg back in and repeat. **Progression:** Straighten leg of study hip; add ankle cuff weight; increase exercise band resistance (yellow through to blue).
Bridging	Weight bearing	Bridge	**Instruction:** Lie on a firm surface with knee bent and feet flat on surface. Place feet hip-width apart. Lift buttocks from the surface. Hold for 2–3 s then slowly lower. **Progression options:** Add weight.
	Weight bearing	Split leg bridge	**Instruction:** Lie on a firm surface. Place feet hip-width apart. Move study leg closer to buttocks and towards centre. Lift buttocks, taking more weight through study leg. Hold for 2–3 s then slowly lower.
	Weight bearing	Double to single leg bridge	**Instruction:** Lie on a firm surface with knees bent and feet flat on surface. Squeeze buttocks and lift buttocks from surface. Keep hips level, lift non-study leg off surface. Hold for 2–3 s. Ground non-study leg and slowly lower buttocks onto support surface. **Progression options:** Increase duration of the hold; move onto advance single leg bridge with non-study leg of the support surface throughout exercise.
**Functional / Knee Extensors**			
**Functional exercises**	Weight bearing	Partial squats	**Instruction:** Standing with feet shoulder-width apart. Bend hips and knees. Lower down slightly. Hold for 2–3 s and slowly straighten back up.
	Weight bearing	Partial squats against wall	**Instruction:** Lean back against wall and step feet hip-width apart, about 40 cm away from the wall. Slide down wall, stopping before knees go past toes. Hold for 3 s, and slowly slide back up. **Progression:** Add resistance band around knees; half-way holds (hold for 2–3 s at the halfway point while going up and/or down).
	Weight bearing	Split leg wall squats	**Instruction:** Lean back against wall and step feet hip-width apart, about 30 cm away from the wall. Move your non-study leg a further 15 cm away from the wall. Slide down wall, stopping before knees go past toes. Hold for 2–3 s, and slowly slide back up.
	Weight bearing	Sit to stand	**Instruction:** Sit on a chair with feet shoulder-width apart. Slowly stand until fully straight and sit down slowly. **Progression:** Add a resistance bank around your knee; half-way holds (hold for 3 s at the halfway point while going up and/or down); use a lower chair; hold a weight.
	Weight bearing	Sit to stand with more weight on study leg	**Instruction:** Sit on a chair with feet shoulder-width apart. Take more weight on study leg by either (a) placing non-study leg further forward so that study leg is closer, or (b) shifting both feet sideways so study leg is lined up with middle of body. Push through the heel of study leg and stand up without using hands. Slowly sit down.
	Weight bearing	Step ups	**Instruction:** Place study leg on a step. Push through the heel of study leg and bring up other leg. Lightly touch the non-study foot onto the step, then step it back down to the starting position. **Progression:** Use higher step; hold a weight.
**Knee Extensors**	Non-weight bearing	Inner range quads over roll	**Instruction:** Put a rolled-up towel under your knee of study leg. Keep the knee cap and toes pointing toward the roof. Keeping the back of the knee in contact with the towel, push the back of your knee down into the towel and straighten your study leg. Hold for 5 s and slowly lower down.
	Non-weight bearing	Seated knee extension	**Instruction:** Sit on a firm chair. Slowly lift study leg foot and straighten knee until fully straight. Hod for 5 s and slowly lower. **Progression:** Tie resistance band in a loop, and place around leg of the chair. Sit in chair and place your leg into the loop, with back around front of the foot. Increase exercise band resistance (yellow through to blue); add ankle cuff weight.
**Hip Abductor Exercises**	Weight bearing	Standing side leg raises	**Instruction:** Standing with resistance band around ankles. Lift study leg out to side, leading with heel. Hold for 1–3 s then lower slowly. **Progression:** Increase exercise band resistance (yellow through to blue).
	Weight bearing	Standing leg wall press	**Instruction:** Stand sideways with non-study leg against a wall. Lift the non-study leg off the floor, so that hip, thigh and knee touch the wall. Keep body still and push non-study leg into the wall. Hold 3–5 s then lower slowly.
	Non-weight bearing	Side lying leg raises	**Instruction:** Lie on a firm surface with study leg on top. Bend bottom leg and use arms for support. Slowly raise study leg, keeping knee faceting forwards and avoid rolling backwards. Hold for 1–3 s then lower slowly. **Progression** Add cuff weight on the top leg.
	Weight bearing	Crab walking	**Instruction:** Place resistance band around thighs/knees, and separate legs about 10 cm. Slight bend both knees. Step sideways against the tension of the resistance band, keeping legs apart. Take 3 steps right and 3 steps left. Continue for 30 s to complete one round. **Progression:** Place resistance bands around ankles; incorporate zig-zags; increase exercise band resistance (yellow through to blue).
**Hip Flexor Exercises**	Non-weight bearing	Crook lying hip bends	**Instruction:** Lie on a firm surface with knees bent and feet flat on the floor. Keeping your non-study leg bent, slowly raise the study leg into the air. Slowly lower down again. **Progression:** Add weight to the study leg, just abovet the knee; straighten the knee.
	Non-weight bearing	Face up lying hip bends off edge of the bed	**Instruction:** Lie on a firm surface, with your study leg hanging off the end of the bed. Bring the knee of the non-study leg to chest and hold with arms. Tuck your tail under and keep your back flat against the bed. Slowly raise your study leg into the air keeping the knee bent. Slowly lower study leg back down to bed level, no further. **Progression:** Add weight to the study leg, just about the knee; straighten the knee.
	Weight bearing	Standing knee raises	**Instruction:** Stand with feet shoulder-width apart. Use a table or chair for balance. Bend study leg up so that thigh is parallel with the ground. Slowly lower back down. Touch foot lightly with the ground before repeating.
**Hip Adductor Strengthening Exercises**	Non-weight bearing	Crook lying leg squeeze with 5 s hold	**Instruction:** Lie on a firm surface with knee bent and feet on the floor. Place a ball or cushion between knees. Keep tail tucked under. Gently squeeze knees together and build to a moderate pressure. Hold for 5 s, then relax.
	Weight bearing	Standing resistance band adduction	**Instruction:** Tie a resistance band to a stable support and loop the band around the study leg ankle. Step away from the support to create tension in the band. Move your study leg to the midline, pulling against the resistance bank. Keep all your weight supported on your non-study leg. Slowly return your study leg to the starting position. **Progression option:** Increase resistance (yellow through to blue).
	Non-weight bearing	Side lying hip adduction	**Instruction:** Lie on your side with the non-study leg on top. Place your non-study foot in front of other knee. Straighten study leg, with knee facing forward. Keep body still and slowly life the study leg up and off the bed/floor. Slowly lower back down. **Progression:** Add weight to the study leg.
**Arm Strengthening Exercises**			
**Arm flexors**	Non-weight bearing	Bicep curls	**Instruction:** Stand with back straight and arms by side. Hold a weight (around 500 g) in each hand. Bend one elbow and bring hands towards shoulder, then lower slowly down. Repeat on the other side. **Progression:** Increase resistance.
**Arm extensors**	Weight bearing	Wall push up	**Instruction:** Stand facing a wall with feet shoulder-width apart about 30 from the wall. Place palms on the wall at shoulder height. Slowly bend elbows and lean towards the wall. Slowly straighten elbows to return to starting position. **Progression:** Increase repetitions.

### Diet

Participants in the diet and exercise group will also receive six individual consultations with a dietitian via videoconference over 6 months. The timing of these consultations is recommended in weeks 1, 3, 6, 10, between 14 and 17 weeks, and between 19 and 23 weeks, but will be negotiated between the participant and their dietitian. The first consultation will occur 2–3 days following the first consultation with the physiotherapist and will last for approximately 45 min. The remaining consultations will last approximately 30 min. For each participant, dietitians will individualise a dietary intervention involving a ketogenic VLCD. Our pilot study using a ketogenic VLCD demonstrated high acceptability among people with hip OA [22]. Participants will be encouraged to lose at least 10% of their body weight. Five percent weight loss is recommended for improvement in OA symptoms [8] albeit based on trials in knee OA but a dose response is apparent with greater benefits found for 10% or more weight loss. We have demonstrated that 10% weight loss is achievable in our pilot study using a ketogenic VLCD [22].

Prior to their first consultation, participants will receive via post a variety of Optifast meal replacements (Nestlé Health Science, Rhodes, Australia) equivalent to two meals per day for 1 month and educational materials to support weight loss (Table 1). Participants will receive their preferred Optifast meal replacements over the subsequent 5 months. Participants will be asked to weigh themselves at home weekly to record their weight in their logbook throughout the duration of the trial. The purpose of tracking weekly weight is help to motivate participants.

During the first consultation, a personalised management plan will be developed, including appropriate weight loss goals and target weight. Participants will be asked to replace two meals each day, with Optifast meal replacement products and consume a low-carbohydrate meal consisting of protein (e.g. meat, seafood, eggs or tofu), non-starchy vegetables or salad and a tablespoon of oil/fat (if gall bladder in situ). The diet consists of approximately 800 kcal (3280 kJ) per day. Modifications of the diet will be permitted as necessary. Throughout the remaining five consultations, dietitians will discuss progress and use motivational interviewing principles and techniques to help motivation, self-efficacy and overcome barriers preventing participants completing their weight loss plan. Dietitians will also discuss the resource booklets sent to participants, to help adhere to their weight loss plan such as choosing a support person and planning for unforeseen events.

Once participants achieve at least 10% weight loss, they will choose whether to transition to a weight maintenance phase or continue with the ketogenic VLCD to reduce weight further. The transition phase will last at least 2 weeks and involves reducing meal replacements to one meal per day and reintroducing low glycaemic index carbohydrates. Upon completion of the transition phase, participants will enter the maintenance phase and be asked to consume a healthy diet in accordance with the Commonwealth Scientific and Industrial Research Organisation Total Wellbeing diet of high protein, low glycaemic index carbohydrate and low fat. Participants who regain a minimum of 2 kg or more will be advised to again engage in the ketogenic VLCD for 1–2 weeks. Online consultation notes will be used by dietitians. Participants will have access to meal replacements from enrolment to 6 months at no cost to themselves, after which if they opt to re-engage with the ketogenic VLCD, meal replacements will be self-funded. Long term weight maintenance strategies will be addressed during the program to help participants maintain their weight loss after their dietitian support finishes.

### Physiotherapist and dietitian training

Eight musculoskeletal physiotherapists with experience managing people with hip OA, and current registration with Australian Health Practitioner Regulation Agency will deliver the exercise program. All physiotherapists attended a two-hour training session and were provided with a treatment manual describing the exercise program. Five accredited dietitians, with clinical experience in weight management will deliver the diet intervention. All dietitians will complete i) training in best-practice OA management delivered by researchers; ii) motivational interviewing skills delivered by Health and Wellbeing Training Consultants over 2-days; iii) ketogenic VLCD delivered by researchers via 1-h webinar. Further details on training have been published elsewhere [28]. Telephone meetings will be conducted between the research team, physiotherapists and dietitians to address any study issues that arise.

### Treatment fidelity

Throughout the trial, clinicians will be encouraged to discuss any issues that arise delivering the interventions with the researchers. Treatment notes from consultations will be assessed for dietitian and physiotherapist adherence to trial protocol.

### Outcome measures

Table 3 summarises the outcome measures and assessment time-point for each outcome measure. The primary outcome is overall average hip pain severity in the last week measured on an 11-point numeric rating scale (NRS), where 0 = ‘no pain’ and 10 = ‘worst pain possible’ [34] measured at baseline, 6 months and 12 months. This is a validated measure of pain that has been recommended for use in OA clinical trials [35]. Conclusions regarding efficacy will be based on the 6-month change in our primary outcome.

**Table 3:** Outcome summary

Domain	Instrument	Timepoint
**Descriptive measures**		Baseline
Age, sex, height		Baseline
Duration of hip symptoms		Baseline
Geographical location		Baseline
Education level		Baseline
Current employment status		Baseline
Problem in other joints		Baseline
Radiographic disease severity	Kellgren and Lawrence grading scale	Baseline
Comorbidities	Self-Administered comorbidity questionnaire	Baseline
Treatment expectation	5-point ordinal scale	At group allocation
**Primary outcome**		
Overall average hip pain in past week	11-point NRS	Baseline, 6 and 12 months
**Secondary outcomes**		
Weight	Self-reported	Baseline, 6 and 12 months
Body mass index		Baseline, 6 and 12 months
Visceral fat mass	Dual energy x-ray absorptiometry	Baseline and 6 months
Total body fat mass	Dual energy x-ray absorptiometry	Baseline and 6 months
Hip pain	11-point NRS	6 and 12 months
Hip pain, difficulty with activities of daily life, hip-related quality of life	HOOS	Baseline, 6 and 12 months
Health-related quality of life	AQoL-8D	Baseline, 6 and 12 months
Perceived change in physical activity since baseline	7-point ordinal scale	6 and 12 months
Perceived change in hip problem since baseline	7-point ordinal scale	6 and 12 months
**Other measures**		
Total body lean mass	Dual energy x-ray absorptiometry	Baseline and 6 months
Depression, anxiety and stress	Depression, Anxiety and Stress Scale	Baseline, 6 and 12 months
Pain-related fear of movement	Brief Fear of Movement Scale	Baseline, 6 and 12 months
Self-reported medication use		Baseline, 6 and 12 months
Self-reported use of co-interventions		6 and 12 months
Self-control for eating	Weight Efficacy Lifestyle Questionnaire	Baseline, 6 and 12 months
**Adherence**		
Self-rated adherence to physical activity plan	5-point Likert scale	Baseline to 6 months
Self-rated adherence to weight management	5-point Likert scale	Baseline to 6 months
Self-rated number of strengthening exercise sessions completed over past 2 weeks		6 months
Number and duration of consultations with physiotherapist		Baseline to 6 months
Number and duration of consultations with dietitian (diet and exercise group only)		Baseline to 6 months
Number of weeks using meal replacements (diet and exercise group only)		Baseline to 6 months
**Harms**		
Adverse events		6 and 12 months

Secondary outcomes

i) Body weight, measured in kilograms. Participants will be asked to use the same set of scales across the time-points;
ii) Body mass index, measured in kg/m^2^;
iii) Visceral fat mass, measured in grams and percentage of total body mass;
iv) Total body fat mass, measured in grams and percentage of total body mass;
v) Hip pain, measured as the proportion who meet or exceed the minimal clinical important difference (MCID) in NRS pain (at least 1.8 units of improvement) [36];
vi) Hip Osteoarthritis Outcome Scale (HOOS) pain subscale, normalised to 0–100 scale (100 indicating no symptoms and 0 indication extreme symptoms) [37];
vii) HOOS activities of daily living subscale, normalised to 0–100 scale (100 indicating no difficulty and 0 indication extreme difficulty) [37];
viii) HOOS quality of life subscale, normalised to 0–100 scale (100 indicating better quality of life and 0 indication lower quality of life) [37];
ix) Health-related quality of life, measured using the Assessment of Quality of Life Instrument (AQoL-8D) [38];
x) Global rating of change in physical activity, scored on a 7-point Likert scale for “change in your physical activity levels since you began the study” from “much less” to “much more”;
xi) Global rating of overall change in hip problem, scored on a 7-point Likert scale for “change in your hip problem since you began the study” from “much worse” to “much better” when compared to baseline.

#### Descriptive measures

Descriptive measures include age; body height, sex; duration of symptoms; geographical location; education level; current employment status; problems such as pain, aching discomfort or stiffness in other joints; radiographic disease severity using the Kellgren and Lawrence scale [39]; comorbidities assessed using the Self-Administered Comorbidity Questionnaire [40]; and treatment expectation assessed on a 5-point ordinal scale.

#### Other measures

Other measures will be collected to describe total body lean mass; depression, anxiety and stress [41]; pain-related fear [42], and self-control for eating [43] (Table 3). These measures will not be used to determine treatment efficacy.

### Treatment adherence

At 6 months, participants will report the number of strengthening exercise sessions performed over the prior 2 weeks. At 6 months, adherence to the prescribed physical activity programs, and weight management plan, if applicable, will be self-reported by participants using a 5-point NRS (0 = none of the time; 5 = all of the time). Physiotherapists and dietitians will record the number of attended consultations and the duration of the consultations throughout the 6-months for each participant. The number of weeks participants use meal replacements (diet and exercise group) will also be recorded.

### Adverse events

Adverse events will be defined as any untoward medical occurrence, irrespective of whether it is related to the study interventions or not. Participants are advised to report any adverse events to their study physiotherapist or dietitian as soon as they can. If necessary, treatment is discontinued and further medical advice is arranged. Clinicians are instructed to report any adverse events to the Trial Coordinator and record the event in their consultation notes. Participants are provided with a “help” email address to contact if they have any issues with their exercise/physical activity or weight management program in between consultations. The email is monitored daily by research staff and, if necessary, staff can seek further advice/guidance from the participant’s physiotherapist and/or dietitian. Adverse events will be acquired from participants at 6 and 12 months. Participants are requested to provide details on the nature of the event, if they sought treatment from a doctor or any other health professional, if the event was life-threatening or required hospitalisation, and whether they considered the event related to any component of the intervention they are receiving as part of the study. The Chief Investigator along with other study investigators will determine causality. If the event is related to the trial, it will be deemed a related adverse event. A serious adverse event will be defined as any untoward medical occurrence that; i) results in death; ii) is life-threatening; iii) requires hospitalisation or prolongation of existing inpatients hospitalisation; iv) results in persistent or significant disability or incapacity; v) is a congenital anomaly or birth defect, or; vi) any other important medical condition which, although not included in the above, may require medical or surgical intervention to prevent one of the outcomes listed.

### Co-interventions

Participants will indicate whether they have taken common arthritis medications over the past month for their study hip at baseline. At 6 and 12 months, participants will indicate whether they used oral opioid medication, paracetamol or paracetamol combinations and oral anti-inflammatories for their study hip ‘less often’, ‘more often’ or the ‘same as before’ (over the past month). At baseline, 6 and 12 months, participants will complete a customised questionnaire regarding prescription and non-prescription medications including injections, over the past 6 months.

### Sample size calculation

We aim to detect a MCID between groups of 1.8 NRS units in change in overall hip pain severity (baseline minus 6 months) [36]. We have performed the sample size calculation taking into account differential clustering between arms. As the physiotherapists treat participants in both arms of the trial, only the clustering by dietitians was accounted for in the sample size calculation. We have assumed a conservative standard deviation at baseline across all participants of 2.5 units and a conservative correlation between baseline and 6-month scores of 0.25, as guided by our other research [44]. We have also assumed an intra-cluster correlation of 0.05 and that there will be 5 dietitians treating approximately 8 patients each. Given these parameters, we need 40 per arm to achieve 80% power to detect the MCID in pain at a 0.05 significance level. Allowing for 20% attrition, we will recruit 50 people per arm in total (*n* = 100).

### Statistical analyses

A biostatistician will analyse blinded data. Main comparative analyses between groups will be performed using intention-to-treat. Multiple imputation will be used if the primary outcome has greater than 5% missing data. For the primary hypothesis, differences in mean change in pain (baseline minus follow-up) will be compared between groups using a mixed-effects linear regression model including all data from 6 and 12 months for each participant with random effects for participants, and accounting for clustering by physiotherapist and dietitian as appropriate. The model will be adjusted for baseline values and the stratifying variables of site (Sydney/Melbourne) and sex. Similar mixed-effects linear regression models will be used for continuous secondary outcomes measured at baseline, 6 months, and 12 months. The proportion of participants in each group that show an improvement that reaches or exceeds the MCID in pain (≥1.8 units) will also be calculated. For this and other binary outcomes, groups will be compared using risk differences and risk ratios, calculated from logistic regression models adjusted for the stratifying variables of site location and sex, and fit using generalized estimating equations to account for clustering. All effect sizes, 95% confidence intervals and *p*-values will be reported and used to interpret the results. Standard diagnostic plots will be used to check model assumptions. A full statistical analysis plan, including a description of secondary analyses (e.g. mediation analyses and/or health economic analysis) will be published prior to undertaking the formal analysis of collected data.

### Timelines

Ethical approval was obtained from the Human Research Ethics Committee of The University of Melbourne in March 2021. Recruitment commenced in May 2021 and will be completed in May 2022, pending COVID-related restrictions. The trial is due for completion in May 2023 when all participants will have completed 12-month data.

### Patient and public involvement

The trial has been endorsed by the Australia and New Zealand Musculoskeletal (ANZMUSC) Clinical Trial Network indicating its high priority and quality, importance to consumers/patients, clinicians and policy makers, and its potential to improve patient outcomes. The ANZMUSC endorsement process included an assessment of the protocol by a scientific and consumer advisory group that supported the design of the trial.

### Dissemination

Study findings will be disseminated through publication in peer-reviewed journals and conference presentations.

## Discussion

This protocol presented the rationale, theoretical foundation, and protocol for a two-arm comparative effectiveness clinical trial comparing diet and exercise to exercise only for people with hip OA. Clinical guidelines recommend weight loss for hip OA in the absence of robust evidence on the effectiveness of diet interventions for hip OA. High quality trials specific to hip OA have been highlighted by leading international organisations as a priority [5]. Findings from the ECHO trial will assist clinicians and patients with hip OA in determining whether a diet intervention in addition to exercise is beneficial for hip OA symptoms compared to exercise alone.

## Supplementary Information

**Additional file 1.** ECHO study protocol version 2.

**Additional file 2.** ECHO study participant consent form.

## Data Availability

The datasets used and/or analyses including statistical code will be available from the corresponding author (k.bennell@unimelb.edu.au) on reasonable request once the study has been completed.

## Abbreviations

ANZMUSC: Australia & New Zealand Musculoskeletal Clinical Trials Network
ANCOVA: Analysis of Covariance
AQoL-6d: Assessment of Quality of Life instrument − 6 dimension
BMI: Body Mass Index
CHESM: Centre for Health, Exercise and Sports Medicine
CI: Confidence Interval
MCID: Minimal Clinically Important Difference
NRS: Numeric Rating Scale
OA: Osteoarthritis
OARSI: Osteoarthritis Research Society International
PLS: Plain Language Statement
RCT: Randomised Controlled Trial
SD: Standard Deviation
VLCD: Very Low-Calorie Diet
