# Supplementary material for: Effects of adding a diet intervention to exercise on hip osteoarthritis pain: protocol for the ECHO randomized controlled trial
Source: BMC Musculoskelet Disord. 2022 Mar 5;23:215. doi: 10.1186/s12891-022-05128-9 (PMC8898434; doi:10.1186/s12891-022-05128-9)
Supplement: Supplementary file 1 — Additional file 1. ECHO study protocol version 2. [file 12891_2022_5128_MOESM1_ESM.pdf]

Effects of a weight loss program in people with hip osteoarthritis: a randomised controlled trial.

Authors
Dr Michelle Hall
Prof Rana Hinman
Dr Priya Sumithran
Mr Nick Murphy
Ms Fiona McManus
Ms Gabby Knox
Ms Libby Spiers
Dr Karen Lamb
Prof Flavia Cicuittini
Prof David Hunter
Prof Steve Messier
Prof Kim Bennell

Sponsor
University of Melbourne

CONFIDENTIAL

This document is confidential and the property of the University of Melbourne.
No part of it may be transmitted, reproduced, published, or used without prior written authorisation from the institution.

STATEMENT OF COMPLIANCE

This document is a protocol for a clinical research study. This clinical trial will be conducted in compliance with all stipulation of this protocol, the conditions of the ethics committee approval, the NHMRC National Statement on ethical Conduct in Human Research (2007 and all updates), the Integrated Addendum to ICH E6 (R1): Guideline for Good Clinical Practice E6 (R2), dated 9 November 2016 annotated with TGA comments.

Contents

STATEMENT OF COMPLIANCE 1

GLOSSARY OF ABBREVIATIONS 5

1. Study Management 5

2. INTRODUCTION AND BACKGROUND 8

2.1 Background Information 8

2.2 Research Question 10

3 STUDY OBJECTIVES 10

4. STUDY DESIGN 10

5. PARTICIPANT ENROLLMENT AND RANDOMISATION 15

5.1 Recruitment 15

5.2 Eligibility Criteria 15

5.2.1 Inclusion Criteria 15

5.2.2 Exclusion Criteria 16

5.3 Informed Consent Process 16

5.4 Enrolment and Randomisation Procedures 17

5.5 Blinding Arrangements 17

5.6 Participant Withdrawal 17

5.7 Trial Closure 17

5.8 Continuation of therapy 17

6. STUDY VISITS AND PROCEDURES SCHEDULE 19

7. DATA COLLECTION 20

8. INTERVENTIONS 20

9. ADVERSE EVENT REPORTING 22

9.1 Specific Safety Considerations (E.g. Radiation, Toxicity) 23

10. STATISTICAL METHODS 23

10.1 Sample Size Estimation 24

10.2 Statistical Analysis Plan 24

10.3 Interim Analyses 24

11. DATA MANAGEMENT 24

11.1 Data Collection & Storage 24

11.2 Data Confidentiality 25

11.3 Study Record Retention 25

12. ADMINISTRATIVE ASPECTS 25

12.1 Independent HREC approval 25

712.2 Participant reimbursement 25
12.3 Financial disclosure and conflicts of interest 26
13. USE OF DATA AND PUBLICATIONS POLICY 26
14. REFERENCES 27

PROTOCOL SYNOPSIS

Title	Effects of a weight loss program in people with hip osteoarthritis: a randomised controlled trial.
Objectives	To compare the effects of a weight loss and exercise program to exercise only on clinical outcomes in people with hip osteoarthritis (OA) and overweight or obesity.<br><br>*Primary objective:* To determine whether a weight loss and exercise program will improve hip pain greater than an exercise program alone at 6 months.
Study Design	A superiority, 2-group, parallel randomised controlled trial (RCT)
Planned Sample Size	100 participants
Selection Criteria	Participants will be aged ≥ 50 years fulfil American College of Rheumatology classification criteria of hip pain and radiographic hip (OA). Other inclusion criteria are history of hip pain ≥3mths; hip pain most days of past month; average hip pain over past week ≥4 on 11-point numerical rating scale (NRS; 0=no pain, 10=worst pain possible); access to a device with internet connection; body mass index >27 kg/m^2; <2 kg weight loss over the previous 3 months; able to give informed consent and participate in the interventions and assessment procedures.
Study Procedures	Following informed consent and baseline assessment, participants will be randomly allocated to receive either i) a weight loss and exercise intervention, or; ii) an exercise-only intervention. Participants will undergo five consultations (30-45 minutes) with a physiotherapist over 6 months for prescription of a home-based strengthening exercise program and physical activity plan (to be conducted independently at home), as well as OA education. Participants in the weight loss and exercise group will also undergo six consultations with a dietitian and undergo a ketogenic very low-calorie diet (VLCD) including meal replacements with an intensive weight loss phase and weight maintenance phase. All consultations with physiotherapists and dietitians will be via video conferencing. Participants will be reassessed at 6 and 12 months post-randomisation.
Statistical Procedures<br>Sample Size Calculation:<br>Analysis Plan:	**Sample Size Calculation:** We aim to detect the minimal clinical important difference in pain intensity (1.8 out 10 units on the numeric rating scale). We assume a between-participant standard deviation of 2.5 and a baseline to follow-up correlation of 0.25. We have also assumed an intra-cluster correlation of 0.05 and that there will be 5 dietitians treating approximately 8 participants each. To achieve 80% power and 5% significance level we require 40 participants per group. Allowing for a 20% loss to follow-up rate, we aim to randomise a total of 100 participants.<br><br>**Analysis Plan:** A biostatistician will analyse blinded data. Main comparative analyses between groups will be performed using intention-to-treat. If there is >5% missing data, multiple imputation will be used. For the primary hypothesis, difference in mean change in pain (baseline minus follow-up) will be compared between groups using a mixed-effects linear regression model including all data from 6 and 12 months for each participant, adjusting for baseline values and the stratifying variables site location (Melbourne and Sydney) and sex. Participants will disclose their sex at birth (male, female or prefer to not say/other)

	during baseline assessment. Those who select “prefer not to say/other” will be randomised into either the male or female strata. A term for month and an interaction between month and randomised group will also be included as fixed effects, with random effects for participants to account for the longitudinal nature of the data, and for physiotherapists and dietitians to account for any clustering by clinician. Similar methods will be used for continuous secondary outcomes measured at baseline, 6 months, and 12 months. For total body fat mass (measured only at baseline and 6 months), a linear regression model will be used, adjusted for baseline values and the stratifying variables of site location and sex. The proportion of participants in each group that show an improvement that reaches or exceeds the MCID in pain (≥1.8 units) will also be calculated. For this and other binary outcomes, groups will be compared using risk differences and risk ratios, calculated from logistic regression models adjusted for the stratifying variable of site location and sex and fit using generalized estimating equations to account for clustering. Standard diagnostic plots will be used to check model assumptions. Potential mediation by pre-specified variables will be assessed using causal mediation analysis.
Duration of the study	Each participant will be involved for 12 months.

GLOSSARY OF ABBREVIATIONS

ABBREVIATION	TERM
ANZMUSC	Australia & New Zealand Musculoskeletal Clinical Trials Network
ANCOVA	Analysis of Covariance
AQoL-6d	Assessment of Quality of Life instrument – 6-dimension
BMI	Body Mass Index
CHESM	Centre for Health, Exercise and Sports Medicine
CI	Confidence Interval
CRP	c-reactive protein
IL-6	interleukin-6
IL1-Sr	interleukin soluble receptors
MCID	Minimal Clinically Important Difference
NICE	National Institute for Health and Care Excellence
NHMRC	National Health and Medical Research Council
NRS	Numeric Rating Scale
OA	Osteoarthritis
OARSI	Osteoarthritis Research Society International
PASE	Physical Activity Scale for the Elderly
PLS	Plain Language Statement
RACGP	Royal Australian College of General Practitioners
RCT	Randomized Controlled Trial
SD	Standard Deviation
TNF-alpha	tumor necrosis factor-alpha
VLCD	Very Low-Calorie Diet
VAS	Visual Analogue Scale
WOMAC	Western Ontario and McMaster Universities Osteoarthritis Index

1. Study Management

1.1 Principal Investigator

Name	Role	Contact information
Prof Kim Bennell	Prof Bennell is a research physiotherapist and Director of the Centre for Health, Exercise and Sports Medicine (CHESM). She will lead the RCT, manage the budget, and supervise the Trial Coordinator. She will be responsible for publishing the trial protocol and the findings of the trial.	P: 03 8344 4135<br>E: k.bennell@unimelb.edu.au<br>Centre for Health Exercise and Sports Medicine<br>Department of Physiotherapy<br>School of Health Sciences<br>University of Melbourne VIC 3010

1.2 Associate Investigators

Name	Role	Contact information
Prof Rana Hinman	Prof Hinman is a research physiotherapist who has conducted >20 RCTs in musculoskeletal conditions, mostly in OA. She provides osteoarthritis and clinical trials expertise.	P: 03 8344 3223<br>E: ranash@unimelb.edu.au<br><br>Centre for Health, Exercise and Sports Medicine<br>Department of Physiotherapy<br>School of Health Sciences<br>University of Melbourne VIC 3010
Dr Michelle Hall	Dr Hall is an exercise scientist with a special interest in the use of non-pharmacological treatments including exercise and weight loss for people with OA. She will assist the Principal Investigator and Trial Coordinator to conduct the trial.	P: 03 8344 0556<br>E: halm@unimelb.edu.au<br><br>Centre for Health, Exercise and Sports Medicine<br>Department of Physiotherapy<br>School of Health Sciences<br>University of Melbourne VIC 3010
Dr Priya Sumithran	Dr Sumithran is a clinician researcher who leads the Obesity Research Group in the Department of Medicine (Austin). She is also an endocrinologist at Austin Health where she is Head of Obesity Medicine. She brings to the project her expertise in management of obesity.	P: 03 9496 2375<br>E: priyas@unimelb.edu.au<br><br>Department of Medicine<br>University of Melbourne VIC 3010
Dr Nick Murphy	Dr Murphy is an orthopaedic registrar at Central Coast Local Health District in NSW. He brings expertise in imaging and will grade the hip x-rays.	P: 04 0020 5454<br>E: nmur6094@uni.sydney.edu.au<br><br>Institute of Bone and Joint Research, Kolling Institute, University of Sydney.
Prof Flavia Cicuttini	Prof Cicuttini is a rheumatology clinician researcher who leads a multidisciplinary research team at Monash University, with expertise in hip OA.	E: flavia.cicuttini@monash.edu.au<br><br>Clinical Epidemiology<br>Monash University VIC 3010
Prof David Hunter	Prof Hunter is a rheumatology clinician researcher who leads a multidisciplinary research group at University of Sydney. He brings to the project his expertise in hip OA and clinical trials.	P: 02 9463 1887<br>E: david.hunter@sydney.edu.au<br><br>Institute of Bone and Joint Research, Kolling Institute, University of Sydney

Prof Stephen Messier	Prof Messier is Director of the J.B Snow Biomechanics Laboratory at Wake Forest University. He has extensive expertise in the conduct of several large clinical trials specifically on diet and exercise for OA. He brings to the project his expertise in clinical trials and OA.	E: messier@wfu.edu.au<br><br>J.B Snow Biomechanics Laboratory<br>Dept. of Health & Exercise Science<br>Wake Forest University<br>USA
Ms Fiona McManus	Ms McManus is a biostatistician. She will oversee all statistical analyses related to the trial and will assist with writing up of the trial findings.	E: fmcmanus@unimelb.edu.au<br><br>Centre for Epidemiology and Biostatistics<br>Melbourne School of Population and Global Health<br>Level 3, 207 Bouverie Street<br>Melbourne VIC 3010
Dr Karen Lamb	Dr Lamb is a biostatistician and senior research fellow. She will oversee all statistical analyses related to the trial and will assist with writing up of the trial findings.	E: klamb@unimelb.edu.au<br><br>Centre for Epidemiology and Biostatistics<br>Melbourne School of Population and Global Health<br><br>Methods and Implementation Support for Clinical Health Research Platform<br>Faculty of Medicine, Dentistry and Health Sciences<br><br>Level 3, 207 Bouverie Street<br>Melbourne VIC 3000
Ms Gabby Knox	Ms Knox is a research scientist with a background in nutrition, who will take on the role of Trial Coordinator. This will include dietitian and physiotherapist recruitment, participant recruitment & appointment scheduling, administration of all primary and secondary outcome measures, database management.	P: 03 9035 4138<br>E: gabby.knox@unimelb.edu.au<br><br>Centre for Health Exercise and Sports Medicine<br>Department of Physiotherapy<br>School of Health Sciences<br>University of Melbourne VIC 3010
Ms Libby Spiers	Ms Spiers is a research physiotherapist, who will assist to establish and coordinate the trial. She will assist with organising contracts with clinicians and assessment clinics. Other duties will include assisting with ongoing trial governance and recruitment.	P: 03 9035 3886<br>E: libby.spiers@unimelb.edu.au<br><br>Centre for Health Exercise and Sports Medicine<br>Department of Physiotherapy<br>School of Health Sciences<br>University of Melbourne VIC 3010

1.3 Statistician

Ms Fiona McManus	E: fmcmanus@unimelb.edu.au<br><br>Centre for Epidemiology and Biostatistics<br>Melbourne School of Population and Global Health<br>Level 3, 207 Bouverie Street<br>Melbourne VIC 3010
Dr Karen Lamb	E: klamb@unimelb.edu.au<br><br>Centre for Epidemiology and Biostatistics<br>Melbourne School of Population and Global Health<br><br>Methods and Implementation Support for Clinical Health Research Platform<br>Faculty of Medicine, Dentistry and Health Sciences<br><br>Level 3, 207 Bouverie Street<br>Melbourne VIC 3000

1.4 Internal Trial Monitoring Committee

The Principal Investigator, the Trial Coordinator, and at least one other Investigator will meet fortnightly to monitor progress of the trial, including any reported adverse events.

1.5 Sponsor

The University of Melbourne.

1.6 Funding and resources

National Health and Medical Research Council (NHMRC) Investigator Grant (#1174431) and Centre of Research Excellence scheme (#1079078).

2. INTRODUCTION AND BACKGROUND

2.1 Background Information

Osteoarthritis (OA) is a leading cause of pain and disability worldwide[1]. Around 2.1 million Australians (1 in 11 people) have OA, with a 58% increase expected by 2032 due to population ageing and rising obesity rates.[2] Hip OA affects one in four adults over their lifetime[3], and substantially reduces quality of life. In 2012, Australian healthcare expenditure on OA was $3.75 billion[4], of which cost related to hip joint replacements was a substantial contributor. There is no cure for OA and rates of joint replacement are staggering, with a life-time risk of hip replacement for hip OA as high as 1 in 7 for women and 1 in 10 for men[5]. In 2018, 77% of the total hip joint replacements performed in Australia were for individuals with hip OA with pre-obesity or obesity[6]. Treatments that effectively improve symptoms and delay the need for joint replacement are critical. Importantly, the most frequently asked question by people with hip or knee OA, is 'what can I do myself to decrease my symptoms?'[7].

Clinical guidelines[8-12] for hip OA management recommend exercise as the core treatment for symptoms. Systematic reviews and meta-analysis of clinical trials evaluating land-based exercise in hip OA report small-to-moderate benefits for pain and physical function[13, 14]. Muscle weakness is common in hip OA[15] and the majority of evidence for exercise is based on predominately strengthening-related exercise programs[13]. In addition to strengthening exercise, regular physical activity is important as people with OA are at increased of risk of death due to cardiovascular disease[16]. Indeed, many people with hip OA

do not meet physical activity recommendations[17]. There is some evidence that supports the use of exercise to delay hip joint replacement in people with hip OA[18]. However, up to 85% of people with hip OA have overweight or obesity[19] and exercise alone is unlikely to be sufficient to meaningfully reduce symptoms and delay the need for joint replacement in individuals with overweight or obesity. The window for effective non-surgical management is short, with ~20% of patients undergoing a hip replacement one year following initial consultation with OA specialists[20].

Clinical guidelines provide conflicting recommendations about weight loss for hip OA. Due to the absence of clinical trials evaluating the effectiveness of weight loss on hip OA symptoms, the 2019 Osteoarthritis Research Society International guidelines for hip OA management do not recommend weight loss for hip OA[9]. Other clinical guidelines[8, 10-12] including the 2019 American College of Rheumatology guidelines[12], do recommend weight loss for hip OA based on evidence from clinical trials in knee OA. A reduction in body weight by at least 5% is recommended for improvement in clinical and mechanistic outcomes[12]. Irrefutable health benefits are associated with weight loss for those with overweight or obesity, but our systematic review and meta-analysis of clinical trials found no significant effect of weight loss interventions alone on knee OA pain[21]. It remains uncertain whether weight loss in addition to exercise and regular physical activity is superior to exercise alone for hip OA symptoms.

Despite no empirical evidence in hip OA, weight loss may enhance the effects of exercise and regular physical activity on symptoms by reducing hip joint loads and improving levels of inflammatory cytokines. People with end-stage hip OA awaiting hip joint replacement have lower peak hip joint moments compared to healthy controls[22], which is thought to reflect a movement strategy to avoid painful compression of the osteoarthritic hip joint during walking. Joint loads are heavily influenced by body weight, as evidenced by a dose-response relationship between weight loss and lower resultant knee joint forces[23]. Higher systemic concentrations of pro-inflammatory cytokines are associated with pain intensity in people with OA[24]. The effects of weight loss and exercise on inflammatory markers in people with knee OA is minimal, with only a tendency towards a small reduction in c-reactive protein (CRP) identified in our systematic review of knee OA clinical trials[21]. Nevertheless, improvements in inflammatory cytokines including interleukin-6 (IL-6), tumor necrosis factor-alpha (TNF-alpha), interleukin soluble receptors (IL-1sR) and CRP have been collectively demonstrated to partially mediate the causal effect of a diet and exercise intervention on pain and physical function in knee OA[25]. Evidently, much of the evidence is based on knee OA and given the differences between hip and knee OA it is uncertain whether weight loss in addition to exercise and regular physical activity will improve hip pain through similar pathways.

Among the myriad of weight loss strategies, the ketogenic very low calorie diet (VLCD) is promising and leads to greater short-term weight loss compared to low-fat diets[26] and low calorie diets[27]. The ketogenic VLCD is a protocolled plan that is typically characterised by an intensive weight loss phase followed by a transition and maintenance phase. A systematic review and meta-analysis of ketogenic VLCDs found an average 14% reduction in body weight loss in studies with a ketogenic phase up to 12 weeks with loss in body weight stable up to two years follow-up[27]. Additionally, a ketogenic VLCD was associated with improvements in factors related to cardiovascular disease such as blood pressure, total cholesterol and triglycerides. Importantly, using nutritionally complete meal replacement products is considered safe in adults aged up to 80 years or older[28]. We have recently completed a pilot study (n=18) partly funded by the ANZMUSC Clinical Trials Network demonstrating feasibility of a weight loss and exercise intervention delivered remotely. We observed an average of 10% [95% CI -12% to -8%] reduction in body weight by 3 months and no adverse events related to the weight loss program were reported.

It is important to consider the method of delivering health care services to ensure accessibility and scalability of care. This is highlighted more than ever during the global COVID-19 pandemic. Evidence supports the use of telehealth by health professionals, such as physiotherapists and dietitians to reduce knee pain[29] and body weight[30], respectively. Telehealth is endorsed by national physiotherapy[31] and dietitian professional organisations[32] and is acceptable to people with OA[33, 34], dietitians and physiotherapists[33, 35]. In our pilot study, consultations between clinicians (physiotherapists and dietitians) and participants were conducted via videoconferencing platform over 6 months, and the

majority of participants perceived the intervention as extremely satisfactory. The bespoke intervention originally developed for people with knee OA[36], is underpinned by behaviour change theory to empower individuals to self-manage symptoms by reducing body weight and engaging in regular exercise.

This clinical trial proposal underwent independent review by the ANZMUSC Clinical Trials Network, which included a written submission and verbal presentations in an open form to ANZMUSC members and review by two members from each of the Scientific Advisory Committee and Consumer Advisory Group. This trial proposal was endorsed by ANZMUSC, indicating its high priority and quality, potential to improve patient outcomes, importance to consumers/patients, clinicians and policy makers.

2.2 Research Question

Is a 6-month weight loss program in addition to exercise more effective for improving pain than exercise alone in people with hip OA and overweight or obesity?

3 STUDY OBJECTIVES

3.1 Primary Objective

To determine whether a weight loss and exercise program will improve hip pain more than an exercise program alone at 6 months.

3.2 Secondary Objectives

i) To determine whether weight loss and exercise will improve hip pain more than exercise at 12 months.
ii) To determine whether weight loss and exercise will improve other clinical outcomes (physical function, body weight; body fat mass; hip-related quality of life; health-related quality of life; global rating of change in physical activity) at 6 and 12 months.
iii) To evaluate potential mediators of treatment effect on pain, based on the following a priori hypotheses:
   a. Body mass index
      Hypothesis- Effect of the weight loss and exercise intervention on pain compared to exercise only will be mediated by reduced body mass index.
   b. Fat mass
      Hypothesis- Effect of the weight loss and exercise intervention on pain compared to exercise only will be mediated by reduced fat mass.

4. STUDY DESIGN

4.1 Type of Study

A multi-site, two-arm, parallel, pragmatic, superiority RCT will be conducted.

4.2 Study Design

Good Clinical Practice guidelines will be followed. Findings will be reported in an accordance with CONSORT guidelines for non-pharmacological interventions[37], and the Australia & New Zealand Musculoskeletal (ANZMUSC) Clinical Trial Network governance and publication policies[38]. Interventions will be reported according to the TIDIeR checklist[39]. The trial will be prospectively registered and the protocol published following SPIRIT statement[40].

A total of 100 participants with a diagnosis of hip OA (see selection criteria below) will be recruited from metropolitan Melbourne and Sydney via advertisements, print/radio/social media, clinicians and our volunteer database. Participants will be randomly allocated to receive either i) a weight loss and exercise intervention or; ii) an exercise intervention, over 6 months.

4.3 Number of Participants

We will recruit 50 participants per treatment arm, therefore 100 participants will be recruited in total.

4.4 Study sites

The study will be conducted at The University of Melbourne.

4.5 Expected Duration of Study

Anticipated participant recruitment start: March 2021
Anticipated data collection end: Jan 2022

We anticipate recruitment for this study will take approximately 5 months (approx. 20 participants per month). Thus, we expect recruitment to take place between March 2021 to July 2021

4.6 Primary and Secondary Outcome Measures (follow-up time-points are relative to randomisation)

Name	Description	Scale	Time-points measured
**Primary Outcome**			
Severity of hip pain[41]	Scored on an 11-point NRS for average hip pain in the last week.	Ranges from 0 to 10; where 0=no pain and 10=worst pain possible.	Baseline, 6 and 12 months
**Secondary Outcomes**			
Body weight	Measured using home scales	Kilograms	Baseline, 6 and 12 months
Body mass index	Calculated from height (baseline) and weight	kg/m^2	Baseline, 6 months, 12 months
Total body fat mass	Dual energy x-ray absorptiometry	Grams and % of total body mass	Baseline and 6 months
Hip pain	Proportion who meet or exceed the minimal clinical important difference in NRS pain (1.8)	Expressed as percentage relative to number of participants allocated to each group.	6 and 12 months
Hip Osteoarthritis Outcome Scale (HOOS) Pain Subscale[42]	Scored using 10 questions regarding hip pain in the last week, with Likert response options ranging from no pain to extreme pain.	Ranges from 0 to 20 and normalised to 0 – 100 scale. Higher scores indicating less pain.	Baseline, 6 and 12 months
Hip Osteoarthritis Outcome Scale (HOOS) Activities of daily living[42]	Scored using 17 questions regarding hip function in the last week, with Likert response options ranging from no dysfunction to extreme dysfunction.	Ranges from 0 to 68 and normalised to 0 – 100 scale. Higher scores indicating less dysfunction.	Baseline, 6 and 12 months
Hip Osteoarthritis Outcome Scale (HOOS) Quality of Life Subscale[42]	Scored using 4 questions regarding quality of life in the last week, with Likert response options ranging from none to extreme.	Ranges from 0 to 16 and normalised to 0 – 100 scale. Higher scores indicating better quality of life.	Baseline, 6 and 12 months
Quality of life (AQoL-8D)[43]	Scored from 35 questions regarding health-related quality of life in the last week.	Ranges from −0.04 to 1.00; higher scores indicate better quality of life.	Baseline, 6 and 12 months
Global rating of change in physical activity	Scored using a 7-point global rating of change Likert scale with response options ranging from “much less” to “much more” when compared to baseline.	Participants indicate they are “moderately more” or “much more” will be classified as increased. All other respondents will be classified as not increased.	6 and 12 months
Global rating of overall change in hip problem	Scored using a 7-point global rating of change Likert scale with response options ranging from “much worse” to “much better” when compared to baseline.	Participants indicate they are “moderately better” or “much better” will be classified as improved. All other respondents will be classified as not improved.	6 and 12 months
Visceral fat mass	Dual energy x-ray absorptiometry	Grams and % of total body mass	Baseline and 6 months
Other/Process measures			

Total body lean mass	Dual energy x-ray absorptiometry	Grams and % of total body mass	Baseline and 6 months
Depression, anxiety and stress[44]	DASS-21 will be used to measure depression, anxiety and stress. Responses range from 0 (did not apply to me) to 3 (applied to me very much or most of the time)	Range 0-42 with higher scores indicating higher levels depression, anxiety and stress.	Baseline, 6 and 12 months
Pain-related fear[45]	Brief Fear of Movement Scale for Osteoarthritis – consists of 6 questions using a 4-point scale.	Scores range from 6 to 24, higher scores indicate greater fear of movement.	Baseline, 6 months and 12 months.
Self-efficacy for eating control [46]	Weight Efficacy Lifestyle (WEL) Questionnaire Short Form.<br>Scored from 8 statements regarding eating control on a 10-point NRS where 0="Not confident at all that I can resist the desire to eat" and 9="Very confident that I can resist the desire to eat". The WEL Short Form is validated to the original 20 item WEL which was based on 5 constructs: negative emotions, availability, social pressure, physical discomfort, and positive activities.	Total scores range from 0-80; higher scores indicate greater eating self-efficacy	Baseline, 6 and 12 months
Duration of consultations	Recorded by dietitians and physiotherapists for each consultation in treatment records	Recorded in minutes	During intervention (0-6 months)
Attendance at consultations	Recorded by dietitians and physiotherapists for each consultation	Number of consultations attended.<br>Number of consultations cancelled/rescheduled.<br>Number of consultations that were a "failed to attend".	During intervention (0-6 months)
Strengthening exercise sessions performed over the past 2 weeks	Self-reported in whole numbers.	Median sessions and as a percentage adherence will be reported.	6 months
Adherence with physical activity plan	Scored on 5-point Likert scale for "How much of the time in the past 6 months did you follow your physical activity plan?""	5-point Likert scale ranging from "none of the time" to "all of the time".	6 months
Adherence with weight loss program (weight loss plus exercise group only)	Self-reported and scored on a 5-point Likert scale for "How much of the time in the past 6 months did you follow your diet plan as it	5-point Likert scale ranging from "none of the time" to "all of the" time..	6 months

	was outlined by your study dietitian?”		
Number of weeks using meal replacements (weight loss plus exercise group only)	Self-reported	Ranges from 0-26	During intervention (0-6 months)
Adverse events	Reported by participants using survey questions.	Number, and type of events and participant-perceived relationship to the research.	, 6, and 12 months
Co-intervention use	Participants complete a custom-developed table of common co-interventions and report whether they have used them in the past 6 months.	Participants who have used a co-intervention once in the past 6 months will be reported as a current user.	6, and 12 months
Medication use	Self-reported<br><br>Participants will complete a table detailing a variety of pain and arthritis medications and supplements and will indicate if they have used them over the previous month.	Medication and supplements that were taken in the past month.<br><br>At 6- and 12-months participants will indicate if they have taken the medication less often, same as before or more often.	Baseline, 6, and 12 months
Health service usage	Participants will complete a custom-developed survey to indicate the frequency of visits to health care providers, use of prescription and over the counter medications injections, hospitalisation and investigative procedures over 6-month intervals.	Number and type of health services used.	Baseline, , 6, and 12 months
**Baseline Descriptive Measures**			
Age	Calculated from date of birth.	Reported in years	Baseline
Body height	Measured using stadiometer at clinics	Meters	Baseline
Sex	Self-reported	Male or female or not disclosed	Baseline
Duration of symptoms	Participants will self-report the total duration of time since their study hip symptoms began.	Reported in years	Baseline
Geographical location	Determined based on residential postcode and classified according to the Australian Standard Geographical Classification (ASGC) Remoteness Structure)	Number and proportion of participants living in major cities, inner regional	Baseline
Education level	Participants will report their education level using a categorical scale with response options <3 years	The number and proportion of respondents for each category will be reported.	Baseline

	of high school; 3 or more years of high school; some education beyond high school; completed tertiary or higher education.		
Employment status	Participants will report their current employment status using a categorical scale with response options currently employed; retired (not due to health reasons); unemployed/student; homemaker; unable to work due to health reasons.	The number and proportion of respondents for each category will be reported.	Baseline
Problems (pain, aching, discomfort or stiffness) in other joints	Self-reported as yes/no at each location.	The number and proportion of respondents for each pain location will be reported.	Baseline
Radiographic OA disease severity	Rated from non-weight bearing x-ray using Kellgren Lawrence scale[47]	The number and proportion of participants with Grade 2 (mild disease severity), 3 (moderate disease) and Grade 4 (severe disease) will be reported.	Baseline
Comorbidities	Reported using the Self-Administered Comorbidity Questionnaire[48].	The number and proportion of participants reporting each comorbidity will be reported.	Baseline
Expectation of treatment outcome	Rated using a 5-point Likert scale with anchors of “no effect at all” to “complete recovery”	The number and proportion of participants selecting each response option will be reported.	At group allocation

5. PARTICIPANT ENROLLMENT AND RANDOMISATION

5.1 Recruitment

Participants will be recruited from the metropolitan Melbourne and Sydney via advertisements, print/radio/social media, clinicians and our volunteer database. Volunteers will initially be screened by an online form, then over the phone by the Trial Coordinator. Volunteers who are not comfortable with online screening may directly call a telephone number and proceed directly to phone screening. For participants with bilaterally eligible hips, the most symptomatic hip will be deemed the study hip with respect to outcome measurement.

5.2 Eligibility Criteria

5.2.1 Inclusion Criteria

Participants will be eligible for the study if they meet the following inclusion criteria:

i) American College of Rheumatology classification criteria with pain in the groin or hip region on most days of the past month and femoral or acetabular osteophytes and joint space narrowing (superior, axial and/ or medial) on x-ray;[49]
ii) aged 50 years or older;
iii) report history of hip pain ≥ 3months;
iv) report an average pain score of at least 4 on an 11-point numeric rating scale (anchored at 0=no pain, 10=worst pain imaginable) over the previous week;

v) access to a device with internet connection;
vi) have a BMI >27 kg/m^2; based on the Royal Australian College of General Practitioners guidelines for obesity management which indicates usage of a very low energy diet to induce rapid weight loss for those with BMI >30 kg/m^2 or those with BMI >27 kg/m^2 who also suffer comorbidities such as OA[50];
vii) willing and able give informed consent and participate fully in the interventions and assessment procedures;
viii) have ability to weigh themselves (e.g. access to scales);
ix) pass the Exercise and Sports Science Australia stage 1 adult pre-exercise screening system[51] or obtain general practitioner clearance for participation in the study.

5.2.2 Exclusion Criteria

i) weight >150 kgs (due to the added complexities of additional nutritional requirements for individuals above this weight);
ii) inability to speak and read English;
iii) on waiting list for/planning back/lower limb surgery or bariatric surgery in next 12 months;
iv) previous arthroplasty on affected hip;
v) recent hip surgery on affected hip (past 6 months);
vi) self-reported inflammatory arthritis (e.g. rheumatoid arthritis);
vii) weight loss of > 2 kg over the previous 3 months;
viii) already actively trying to lose weight by any of the following mechanisms:
   a. using meal replacements for weight loss
   b. being a member of a commercial weight loss program (e.g. weight watchers)
   c. receiving support from another health care professional for weight loss
   d. using any drugs prescribed to aid in weight loss
   e. using structured meal programs for weight loss such as ‘Lite n’ Easy’
ix) unable to undertake ketogenic VLCD without closer medical supervision including self-reported:
   a. diagnosis of Type 1 diabetes
   b. Type 2 diabetes requiring insulin or other medication apart from metformin
   c. warfarin use
   d. stroke or cardiac event in previous 6 months
   e. unstable cardiovascular condition
   f. fluid intake restriction
   g. renal (kidney) problems (unless clearance is obtained from GP, including GP confirmation that estimated glomerular filtration rate >30 $mL/min/1.73m^2$)
x) any neurological condition affecting lower limbs;
xi) pregnancy or planned pregnancy
xii) vegan dietary requirements due to complexity of delivering a nutritionally complete diet within the ketogenic diet regime.

5.3 Informed Consent Process

All potential participants will receive oral and written information about the purposes, potential risks and processes involved in the study from the research staff. In accordance with to the latest revision of the World Medical Association Declaration of Helsinki, informed consent will be obtained from all participants by signing the consent form after understanding the information delivered.

If participants pass the telephone screening process, which will involve a detailed verbal description of the project to ensure that participants are happy to comply with trial procedures, participants will be sent the Plain Language Statement (PLS) and Consent Form in the post or by email. Participants who indicate during phone screening that they suffer with renal dysfunction (or are unsure) and/or fail the Exercise and Sports Science Australia stage 1 pre-exercise screening system[51] questions will be required

to obtain clearance from their general practitioner (and confirmation that their estimated glomerular filtration rate is >30 mL/min/1.73m2 in the case of renal function concerns) to participate in the study.

Participants will be encouraged to phone researchers if they have any questions or concerns regarding the contents of the PLS and/or Consent Form. After reading the PLS, and if they give their consent to participate, consent will be obtained online using REDCap prior to completion of the baseline questionnaire or they will sign a paper-based consent form and return it via a reply-paid envelope in the post or by scanning and emailing the document to the Trial Coordinator.

5.4 Enrolment and Randomisation Procedures

Participants will be enrolled into the study once the informed consent process has been completed, the baseline DEXA scan has been completed and they have completed the baseline questionnaire on the web-based platform (REDCap) or on paper and returned via post. Each participant will receive a unique study ID code, and this will be documented in the participant's record/database in addition to all study documents.

The randomisation schedule will be prepared by the biostatistician (random permuted blocks) stratified by site (two options: Melbourne and Sydney) and sex (two options: Male and Female). Participants allocated to the *Exercise* group will be randomly allocated to a physiotherapist. Participants allocated to the *Exercise plus weight loss* group will be randomly allocated to one of the same physiotherapists as in the *Exercise* group, and to a dietitian. If a physiotherapist or dietitian is unavailable (e.g. sick, on holiday, etc.), participants will be re-randomised to another available clinician. The schedule will be stored on a password-protected website (REDCap) at the University of Melbourne maintained by a researcher not involved in either participant recruitment or administration of primary/secondary outcome measures. Group allocation will be revealed after baseline assessment has been completed.

5.5 Blinding Arrangements

Participants will not be informed about the study hypotheses, until the study is completed, at which time they will be provided a lay summary of study findings. However, the components of each treatment arm will be disclosed during recruitment. As the primary and some of the secondary outcomes are participant-reported, participants are also the assessors. Physiotherapists and dietitians will not be blinded to group allocation or study hypothesis. Research staff administering and entering the participant-reported data will be blinded. Statistical analyses will be performed blinded.

5.6 Participant Withdrawal

As participation in this study is voluntary, participants may withdraw i) from attending their allocated consultations with their study dietitian and/or physiotherapist, and/or; ii) from continuing with scheduled data collection processes, at any point over their 12-month involvement. Participants may choose to stop attending their allocated consultations but continue with some/all aspects of data collection. In order to minimise data loss, research staff will encourage participants to complete primary outcome measures (at a minimum) at the 6-month time frame, over the telephone if necessary. If a participant withdraws from the study, the nature, timing of and reasons for withdrawal will be recorded (provided the participant responds to contact made by the research team). Any data provided up to the point of withdrawal will be kept and used in analyses, unless the participant specifically requests to withdraw all of their data from the study.

5.7 Trial Closure

For each participant, the longest follow-up duration is at 12 months from randomisation. At this point, final assessment will take place and no further data collection or monitoring will occur.

5.8 Continuation of therapy

As participants are permitted to keep their exercise equipment and exercise instructions after their involvement in the study has ended, they may choose whether they wish to continue their exercise program and weight loss program (to those allocated to combined intervention group) or not.

6. STUDY VISITS AND PROCEDURES SCHEDULE

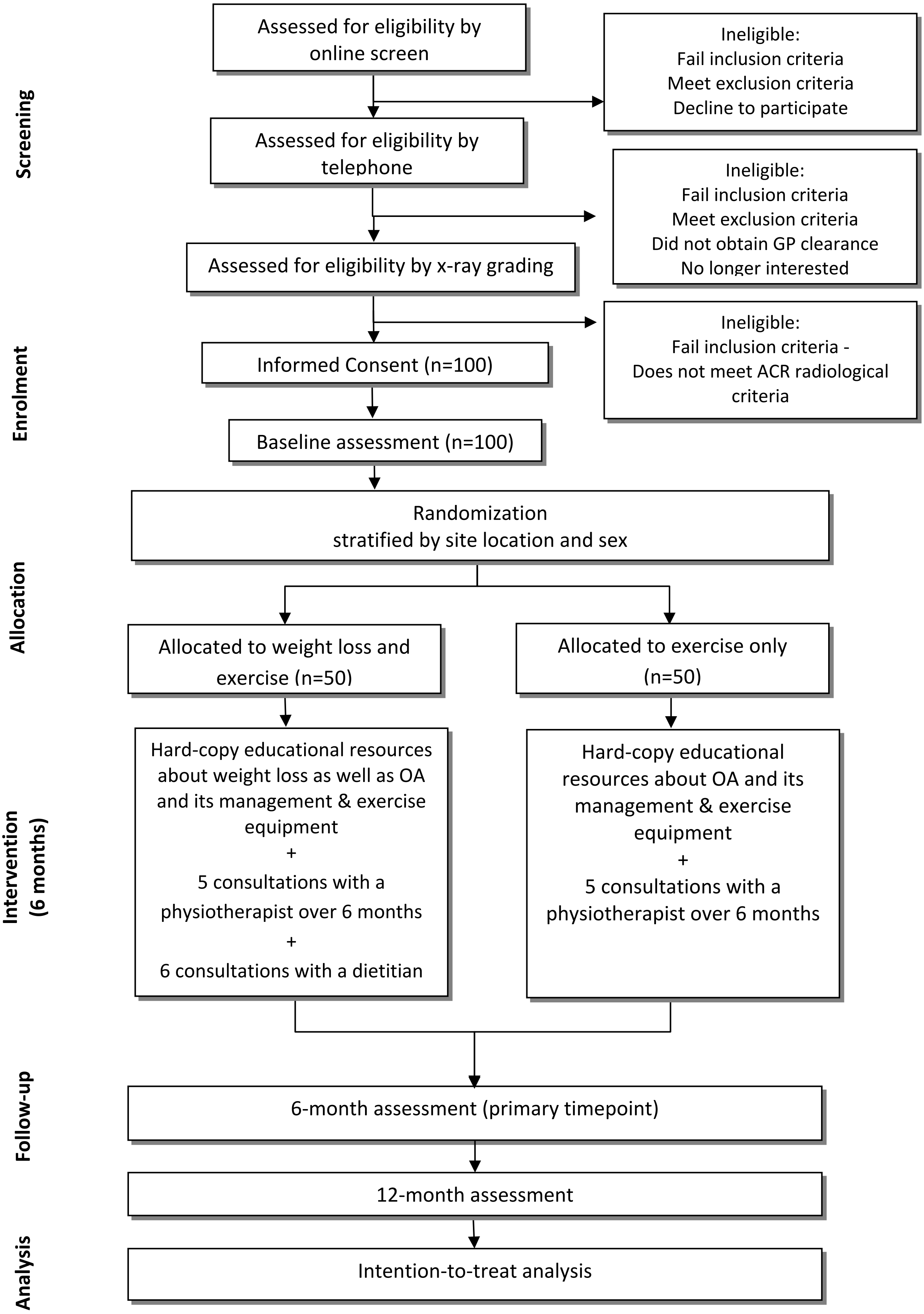

7. DATA COLLECTION

Body composition scans and x-rays will be collected at clinics in Melbourne and Sydney. Other participant-reported or clinician-reported outcomes will be collected electronically via computer or on paper with a pen (if preferred) at all time-points.

8. INTERVENTIONS

Physiotherapists & Dietitians

We will use our networks to recruit practising dietitians and musculoskeletal physiotherapists in Melbourne. The same physiotherapists will deliver exercise programs to both groups. The exercise programs will be protocolised (but allowing some individual variation).

To be eligible to deliver interventions in this RCT, dietitians and physiotherapists and must:

- Hold current accreditation/registration to practice;
- Have an Australian Business Number;
- Be willing to undertake trial training requirements:
- Be willing and available to deliver interventions across the nominated timeframe.

Training

Dietitians will complete training in i) best-practice OA management (half day workshop led by investigators); ii) motivational interviewing skills (2-days training course delivered by Health and Wellbeing Training Consultants who specialise in training clinicians in motivational interviewing; and iii) VLCD (1-hour webinar delivered by research team). Training will occur via online training modules and face-to-face training if permittable. Treatment manuals and copies of patient resources (e.g. diet information brochures for handouts) will be provided to the dietitian in electronic and hard-copy.

Physiotherapists will be trained in trial procedures, best practice OA management, lower limb strengthening and physical activity program, behaviour change techniques to promote adherence and resources for use in the program. Training will occur via online training modules and potentially live training sessions. Treatment manuals and copies of patient resources (e.g. exercise sheets) will be provided to the physiotherapist in electronic and hard-copy.

Participant resources

Each trial participant will be posted a “welcome pack” of resources to facilitate the management plans the physiotherapists will enact during the consultations. These resources will include:

- A welcome letter describing their involvement in the study and listing the resources that they will have received (below);
- 4 information booklets:
    - “Preparing for your consultations”
    - “Osteoarthritis Information”
    - “Exercise Booklet”
    - “Hip Exercise Plan and Log Book””
- 4 coloured therabands (yellow, green, red, blue) for strengthening exercises
- Ankle weights

Participants receiving the weight loss intervention in addition to exercise will also receive the following:

- Educational video about the VLCD
- 4 information booklets:
    - “Weight management how to guide”
    - “Weight management behavioural support activities”
    - “Recipe book”
    - “Food list pocket guide”

- Portion plate

Before the first consultation with the physiotherapist and dietitian (if allocated to receive weight loss and exercise program), participants will complete a pre-consultation survey asking about their main problems and goals, a brief history of their hip symptoms, other health problems, and current physical activity exercise/levels.

Exercise program
This program consists of educational information plus 5 individual video consultations with a physiotherapist over 6-months. The physiotherapist consultations include a structured exercise and physical activity plan and behaviour change support to enhance adherence. Participants will also be given four exercise resistance bands (yellow, green, red, blue) and an ankle weight for home strengthening exercises.

The initial physiotherapy consultation will be approximately 45 minutes long, with follow-up consultations being approximately 30 minutes. Consultations are recommended to occur in weeks 1, 3, 9, 15, and 21, but the precise timing is negotiated between each participant and their physiotherapist.

For the strengthening exercise program, physiotherapists will choose from a list of exercises and aim to prescribe 5-6 exercises, including at least one from each category (i.e. functional/quadriceps strengthening) and an optional extra. The exact number of exercises, as well as sets/repetitions, will be negotiated between the physiotherapist and participant. Intensity is determined using a modified Rating of Perceived Exertion (RPE) scale[52], where it should feel “hard” to “very hard” to perform a full set of each exercises. Participants are encouraged to complete exercises three times per week. For the physical activity plan, physiotherapists encourage the participant to increase their general and incidental levels of physical/aerobic activity based on their individual needs and goals, as well as their current level of activity.

Weight loss and exercise program
Participants in this group will attend a physiotherapist consultation via videoconferencing on 5 occasions and receive the same program as the exercise alone group. Participants will also receive some upper body exercises to maintain muscle mass. In addition, the group will receive 6 individual consultations with a dietitian (over 6 months) also via videoconferencing to help participants undertake a weight loss program. Participants’ first consultation with the dietitian takes place a few days after their first physiotherapist consultation and is approximately 45 minutes in duration, with follow-up consultations approximately 30 minutes. Consultations are recommended to occur in weeks 1, 3, 6, 10, between weeks 14-17 and between weeks 19-23.

Although clinical guidelines suggest that reduction in body weight by 5% can improve clinical and mechanistic outcomes[12], based on evidence from the knee OA literature, our participants will be encouraged to aim to lose at least 10% body weight. The diet program prescribed will be a ketogenic VLCD[53]. The program is based on clinical practice and developed in conjunction with stakeholders including patients, a health insurance company, dietitians and physiotherapists. The diet program comprises two phases: 1) intensive weight loss through a ketogenic VLCD, and 2) transition from ketogenic VLCD onto a longer-term eating plan for weight maintenance. Once participants lose 10% in body weight they can choose whether to commence transition to a weight maintenance phase or continue with the ketogenic VLCD program and aim for further weight loss. Once participants are in a weight maintenance phase, they will monitor their weight and if weight increases by 2kg they will be advised to commence another period of the ketogenic VLCD program using meal replacement products as before but only for 1-2 weeks. The length of time a participant spends in the first phase depends on their weight loss target, their progress and their preference. If a participant does not wish to transition off the ketogenic VLCD diet after 6 months but wants to continue or if they wish to recommence the ketogenic VLCD diet any time between 6 and 12 months, they will need to purchase meal replacements themselves at their own cost. The consultations and support are only available for 6 months after which participants should have the knowledge and skills to self-manage their weight. If participants lose >20% of their body weight within 6 months, the dietitian will refer the participant to see their general practitioner and ensure there are no other health issues.

The ketogenic VLCD diet involves replacing two meals, generally breakfast and lunch, with VLCD products (Optifast meal replacements, or if Optislim is unavailable or the participant is vegetarian). Optislim products provide most of the vitamins, minerals, and metals required for optimal nutrition, and come as bars, shakes, or soups in a variety of flavours. On the diet, one meal (generally dinner) comprises protein (e.g. white or red meat, fish or seafood, eggs, or tofu) and non-starchy vegetables/salad. A small amount (i.e. 1 tablespoon) of fat/oil is also recommended for this meal to stimulate gallbladder contraction. In total, the diet contains 800 calories (3280 kilojoules).

Transitioning off the ketogenic VLCD diet (after target weight is reached) is done by reintroducing foods containing carbohydrates and moving to only one meal replacement per day. This transition phase usually lasts at least two weeks, after which participants commence a healthy eating diet which is consistent with the principles of the Commonwealth Scientific and Industrial Research Organisation (CSIRO) total wellbeing diet (i.e. high protein, low glycaemic index carbohydrate, low fat). Participants are encouraged to weigh themselves regularly thereafter (e.g. at least once per week). Participants who regain 2 kilograms or more are advised to restart the ketogenic VLCD diet with meal replacements for 1-2 weeks.

The trial coordinator will arrange home deliveries of Optifast (or Optislim if Optifast is not available) meal replacement products (shakes, soups or bars) to participants for a maximum of 6 months from the start of the trial. The participants will also be provided with additional educational and behaviour change resources to support their weight loss efforts. Education, information and advice are key components of the intervention to optimally support behaviour change and weight loss. Resources and strategies include:

- setting realistic goals
- keeping a food diary
- monitoring weight weekly
- finding a support person to help
- learning about healthy food choices and portion size
- identifying food triggers
- planning for ‘at risk situations’
- working out barriers and finding ways to overcome them
- changing any negative thought patterns
- engaging in relaxation, mindfulness and distraction techniques
- monitoring hunger levels

Treatment fidelity

Treatment notes from consultations will be assessed be research staff for dietitian and physiotherapist adherence to trial protocols.

9. ADVERSE EVENT REPORTING

Participants who experience any adverse outcomes will be instructed to discuss these with their project dietitian or physiotherapist who will institute appropriate advice and/or changes to the weight loss plan, strengthening exercise program and/or physical activity plan as appropriate. Any risks to participants are likely to be minor and transient. Adverse events will be defined in accordance with The Good Clinical Practice guidelines as any untoward medical occurrence in a clinical trial participant that does not necessarily have a causal relationship with the treatment. Adverse events will be ascertained by survey questions to participants at 6 and 12 months. Based on this information, the Chief Investigator will determine causality. If the event is related to the trial in the opinion of the Chief Investigator, it will be deemed a related adverse event. A serious adverse event will be defined as any untoward medical occurrence that:

1. Results in death,

2. Is life-threatening
3. Requires hospitalisation or prolongation of existing inpatients´ hospitalisation,
4. Results in persistent or significant disability or incapacity,
5. Is a congenital anomaly or birth defect
6. Any other important medical condition which, although not included in the above, may require medical or surgical intervention to prevent one of the outcomes listed.

Due to the low-risk nature of the interventions (weight loss, home-based exercise and physical activity prescribed by dietitians and physiotherapists) in this trial, related serious adverse events (incapacitating, life-threatening, hospitalisation or death) are extremely unlikely but will be reported to the Sponsor's Ethics Committee should they occur. Participants, dietitians and physiotherapists will be advised to report any serious adverse events (incapacitating, life-threatening, hospitalisation or death) to the Trial Coordinator as soon as they can by telephone or email, which will be documented and reported to the HREC as per University policy.

Any adverse events reported by telephone or in questionnaires will be reported to the internal Trial Monitoring Committee, including the Chief Investigator (Prof Bennell) will be responsible for deciding what action, if any, is needed on a case by case basis.

9.1 Specific Safety Considerations (E.g. Radiation, Toxicity)

Participants will be exposed to small amounts of radiation through the acquisition of the DEXA scans (baseline and 6-month follow-up) and hip x-ray (baseline only, and only if the participant has not had a hip x-ray within the past 12 months). As part of everyday living, everyone is exposed to natural occurring background radiation and receives a dose of approximately 2 millisievert (mSv) each year. The additional effective dose participants will receive from entering this trial is approximately 0.6mSV. At this dose level, no harmful effects of radiation have been demonstrated as any effect is likely too small to measure. The risk is believed to be very low.

Exercise program: Whilst it is expected that people following accurate advice, education and information about hip pain would experience improved pain and function, there is a small risk of a transient increase in hip pain, stiffness and swelling or discomfort in other parts of the body due to altered/increased exercise or physical activity. Participants will be asked to discuss this with their study physiotherapist who will adjust the program accordingly, as per usual clinical practice. Participants will also be provided with a "help" email address to contact if they have any issues with their exercise/physical activity or weight management program in between consultations.

Ketogenic/very low energy diet: This can make people feel hungry, fatigued, fuzzy-headed, and have headaches, and either diarrhoea or constipation during the first week. This is to be expected and these symptoms are usually manageable and short-lived. Participants who have hypertension or are on medication will be encouraged to book an appointment with their GP if they are experiencing light-headedness and may need a medication adjustment. The study dietitian may prescribe the participant a calcium/vitamin D supplement if they are concerned about their calcium intake while undertaking the ketogenic/very low calorie diet.

Outcome questionnaires: If the DASS-21 questionnaire identifies a participant with symptoms of depression (scores >20), the Study Coordinator will contact the participant to recommend they seek guidance from their GP. The participant will be provided with a letter that they can present to and discuss with their GP.

10. STATISTICAL METHODS

10.1 Sample Size Estimation

We aim to detect the minimal clinically important difference (MCID) between groups of 1.8 (out of 10) units in pain intensity. Measures will be taken at baseline and 6 months. We have performed the sample size calculation taking into account differential clustering between arms. As the physiotherapists treat participants in both arms of the trial, only the clustering by dietitians has been taken into account for the sample size calculation. We have assumed a conservative standard deviation at baseline across all participants of 2.5 units and a conservative correlation between baseline and 6-month scores of 0.25, as guided by similar studies[54]. We have also assumed an intra-cluster correlation of 0.05 and that there will be 5 dietitians treating approximately 8 patients each. Given these parameters, we need 40 per arm to achieve 80% power to detect the MCID in pain at a 0.05 significance level. Allowing for 20% attrition, we will recruit 50 people per arm in total (n=100).

10.2 Statistical Analysis Plan

A biostatistician (McManus) will analyse blinded data. Main comparative analyses between groups will be performed using intention-to-treat. Multiple imputation will be used if an outcome has greater than 5% missing data. For the primary hypothesis, differences in mean change in pain (baseline minus follow-up) will be compared between groups using a mixed-effects linear regression model including all data from 6 and 12 months for each participant. The model will be adjusted for baseline values and the stratifying variable of site location and sex. Participants will disclose their sex at birth (male, female or prefer to not say/other) during baseline assessment. Those who select “prefer not to say/other” will be randomised into either the male or female strata. A term for month and an interaction between month and randomised group will also be included as fixed effects, with random effects for participants to account for the longitudinal nature of the data, and random effects for physiotherapists and dietitians to account for any clustering by clinician. Restricted maximum likelihood estimation will be applied with the Kenward-Roger correction[55]. Similar mixed-effects linear regression models will be used for continuous secondary outcomes measured at baseline, 6 months, and 12 months. For the continuous secondary outcome measured only at baseline and 6 months (total body fat mass), a linear regression model will be used, adjusted for baseline values and the stratifying variables of site location and sex. The proportion of participants in each group that show an improvement that reaches or exceeds the MCID in pain (≥1.8 units) will also be calculated. For this and other binary outcomes, groups will be compared using risk differences and risk ratios, calculated from logistic regression models adjusted for the stratifying variables of site location and sex, and fit using generalized estimating equations to account for clustering. Standard diagnostic plots will be used to check model assumptions. Potential mediation by the pre-specified variables in Section 3.2 will be assessed using causal mediation analysis.

10.3 Interim Analyses

It is not anticipated that any interim analyses will be performed.

11. DATA MANAGEMENT

11.1 Data Collection & Storage

<u>Identifiable data:</u>

- Screening information and study consent forms will be stored within a secure data collection platform (Qualtrics or REDCap) and accessible only by password to the researchers. If participants prefer to complete consent forms in hard copy, paper consent forms will be stored in locked filing cabinets, separate from a cabinet containing any de-identified data and only accessible to the researchers.
- Details of people screened will be stored electronically in a Microsoft /Excel database, accessible only to the research team and stored securely on password-protected servers.
- X-ray images will be stored electronically at the radiology clinics involved in the study and securely stored and subject to the normal confidentiality guidelines adhered to at each clinic. Researchers will access the x-ray images via log-in to the clinic software and export identifiable images, label with

appropriate identification codes and store securely on password-protected servers. We will not retain x-ray images of the participant who have their own eligible x-rays, these will be mailed back to participants after radiographic severity has been graded (recorded on electronic database). For the x-ray screening assessment, the assessor will complete a screening form in (e.g. REDCap) accessible only to the researcher by password. Data from eligible participants will be exported to Microsoft Excel and stored securely on password-protected servers.

- Body composition data will be stored at the research and radiology clinics involved in the study and securely stored on password-protected servers and subject to the normal confidentiality guidelines adhered at the facility. Researches will access body composition data electronically and export identifiable data, label with appropriate participant codes and store securely on password protected servers.

<u>Re-identifiable/coded data:</u>

- Questionnaires: may be completed on paper or electronically, and will contain only participant codes, and no identifying information. Paper copies will be stored in locked filing cabinets, separate from a cabinet containing any identifiable data and only accessible to the researchers. Electronic copies will be stored in the REDCap website, accessible only to the researchers by password protection. Data from within Qualtrics/REDCap will be exported to Microsoft Excel and other statistical packages used by the researchers for analyses and stored securely on password-protected servers.
- All computer files will be stored on secure and backed-up University servers, accessibly only to the researchers using a password.

11.2 Data Confidentiality

No information which could lead to the identification of a participant will be included in the dissemination of results. Only fully non-identifiable data will be presented when disseminating results.

11.3 Study Record Retention

Data will be retained for 15 years consistent with clinical trial recommendations outlined in section 2.1.1 of the National Health and Medical Research Council's "Australian Code for the Responsible Conduct of Research".

12. ADMINISTRATIVE ASPECTS

The trial will be prospectively registered (ANZ Clinical Trials Registry) and the protocol published in a peer reviewed journal. The trial is endorsed by the Australia & New Zealand Musculoskeletal Clinical Trials Network.

12.1 Independent HREC approval

This study is under review by the University of Melbourne Human Research Ethics Committee (HREC), reference number 20516.

12.2 Participant reimbursement

All participants who are enrolled in the RCT (i.e. provide informed consent and complete baseline assessment) will receive exercise equipment (ankle weights; resistance bands) to use throughout the trial and keep once participation is complete. Participants in the weight loss and exercise group will also receive meal replacements (maximum 2 per day) for up to 6 months from the start of the study. Participants will receive hard copy resources to keep which support their exercise program including OA education material and an exercise booklet. Those in the diet plus exercise group will also receive education booklets to support weight loss, and a recipe book which they can keep once participation is complete.

12.3 Financial disclosure and conflicts of interest

There are no conflicts of interest to declare.

13. USE OF DATA AND PUBLICATIONS POLICY

We will publish a protocol paper prior to completion of the trial. The main trial will be published in an osteoarthritis or general medical journal.

Statistical code may be made available from Ms McManus, upon request from individual researchers.

Data may be made available from Professor Kim Bennell, upon request from individual researchers.

The results of the trial will also be disseminated through avenues such as conference presentations, professional organisations, media, social media and consumer organisations.

14. REFERENCES

1. Cross M, Smith E, Hoy D, et al. *The global burden of hip and knee osteoarthritis: estimates from the Global Burden of Disease 2010 study*: BMJ PUBLISHING GROUP; 2014.

2. Victoria. AO. A Problem Worth Solving. The rising cost of musculoskeletal conditions in Australia. *Arthritis & Osteoporosis Victoria.* 2013.

3. Murphy LB, Helmick CG, Schwartz TA, et al. One in four people may develop symptomatic hip osteoarthritis in his or her lifetime. *Osteoarthritis and Cartilage.* 11/01/November 2010 2010;18(11):1372-1379.

4. Cross M, Smith E, Hoy D, et al. The global burden of hip and knee osteoarthritis: estimates from the Global Burden of Disease 2010 study. *Annals of the rheumatic diseases.* Feb 19 2014;73:1323-1330.

5. Ackerman IN, Bohensky MA, de Steiger R, et al. Lifetime Risk of Primary Total Hip Replacement Surgery for Osteoarthritis From 2003 to 2013: A Multinational Analysis Using National Registry Data. *Arthritis care & research.* Nov 2017;69(11):1659-1667.

6. McAlindon TE, Bannuru RR, Sullivan MC, et al. OARSI guidelines for the non-surgical management of knee osteoarthritis. *Osteoarthritis Cartilage.* 2014;24(14):00016-00018.

7. Claassen A, Kremers-van de Hei K, van den Hoogen FHJ, et al. Most Important Frequently Asked Questions From Patients With Hip or Knee Osteoarthritis: A Best-Worst Scaling Exercise. *Arthritis care & research.* Jul 2019;71(7):885-892.

8. 2018 RACoGPGftn-smohakoMR.

9. Bannuru RR, Osani MC, Vaysbrot EE, et al. OARSI guidelines for the non-surgical management of knee, hip, and polyarticular osteoarthritis. *Osteoarthritis Cartilage.* Nov 2019;27(11):1578-1589.

10. Fernandes L, Hagen KB, Bijlsma JWJ, et al. EULAR recommendations for the non-pharmacological core management of hip and knee osteoarthritis. *Annals of the rheumatic diseases.* 2013;72(7):1125-1135.

11. National Clinical Guideline Centre. Osteoarthritis. Care and management in adults. NICE clinical guideline CG177. London; National Institute for Health and Clinical Excellence.

12. Kolasinski SL, Neogi T, Hochberg MC, et al. 2019 American College of Rheumatology/Arthritis Foundation Guideline for the Management of Osteoarthritis of the Hand, Hip, and Knee. *Arthritis & Rheumatology.* 2020;72(2):220-233.

13. Moseng T, Dagfinrud H, Smedslund G, Østerås N. The importance of dose in land-based supervised exercise for people with hip osteoarthritis. A systematic review and meta-analysis. *Osteoarthritis and Cartilage.* 10/01/October 2017 2017;25(10):1563-1576.

14. Moseng T, Dagfinrud H, Smedslund G, Østerås N. Corrigendum to 'The importance of dose in land-based supervised exercise for people with hip osteoarthritis. A systematic review and meta-analysis' [Osteoarthritis Cartilage 25 (2017) 1563–1576]; 2018, 2018; Great Britain.

15. Loureiro A, Mills PM, Barrett RS. Muscle weakness in hip osteoarthritis: a systematic review. *Arthritis care & research.* Mar 2013;65(3):340-352.

16. Veronese N, Cereda E, Maggi S, et al. Osteoarthritis and mortality: A prospective cohort study and systematic review with meta-analysis. *Semin Arthritis Rheum.* Oct 2016;46(2):160-167.

17. Wallis JA, Webster KE, Levinger P, Taylor NF. What proportion of people with hip and knee osteoarthritis meet physical activity guidelines? A systematic review and meta-analysis. *Osteoarthritis and Cartilage.* 11/01/November 2013 2013;21(11):1648-1659.

18. Svege I, Nordsletten L, Fernandes L, Risberg MA. Exercise therapy may postpone total hip replacement surgery in patients with hip osteoarthritis: a long-term follow-up of a randomised trial. *Annals of the rheumatic diseases.* Jan 2015;74(1):164-169.

19. Reyes C, Leyland KM, Peat G, Cooper C, Arden NK, Prieto-Alhambra D. Association Between Overweight and Obesity and Risk of Clinically Diagnosed Knee, Hip, and Hand Osteoarthritis: A Population-Based Cohort Study. *Arthritis & rheumatology (Hoboken, N.J.).* Aug 2016;68(8):1869-1875.

20. Dabare C, Le Marshall K, Leung A, Page CJ, Choong PF, Lim KK. Differences in presentation, progression and rates of arthroplasty between hip and knee osteoarthritis: Observations from an osteoarthritis cohort study-a clear role for conservative management. *International journal of rheumatic diseases.* 2017;20(10):1350-1360.

21. Hall M, Castelein B, Wittoek R, Calders P, Van Ginckel A. Diet-induced weight loss alone or combined with exercise in overweight or obese people with knee osteoarthritis: A systematic review and meta-analysis. *Semin Arthritis Rheum.* Apr 2019;48(5):765-777.

22. Diamond LE, Allison K, Dobson F, Hall M. Hip joint moments during walking in people with hip osteoarthritis: a systematic review and meta-analysis. *Osteoarthritis Cartilage.* Nov 2018;26(11):1415-1424.

23. Messier SP, Beavers DP, Mihalko SL, et al. The effects of intensive dietary weight loss and exercise on gait in overweight and obese adults with knee osteoarthritis. The Intensive Diet and Exercise for Arthritis (IDEA) trial. *Journal of Biomechanics.* 2020/01/02/ 2020;98:109477.

24. Penninx BW, Abbas H, Ambrosius W, et al. Inflammatory markers and physical function among older adults with knee osteoarthritis. *J Rheumatol.* Oct 2004;31(10):2027-2031.

25. Runhaar J, Beavers DP, Miller GD, et al. Inflammatory cytokines mediate the effects of diet and exercise on pain and function in knee osteoarthritis independent of BMI. *Osteoarthritis Cartilage.* Aug 2019;27(8):1118-1123.

26. Purcell K, Sumithran P, Prendergast LA, Bouniu CJ, Delbridge E, Proietto J. The effect of rate of weight loss on long-term weight management: a randomised controlled trial. *Lancet Diabetes Endocrinol.* Dec 2014;2(12):954-962.

27. Castellana M, Conte E, Cignarelli A, et al. Efficacy and safety of very low calorie ketogenic diet (VLCKD) in patients with overweight and obesity: A systematic review and meta-analysis. *Rev Endocr Metab Disord.* Mar 2020;21(1):5-16.

28. Haywood CJ, Prendergast LA, Purcell K, et al. Very Low Calorie Diets for Weight Loss in Obese Older Adults-A Randomized Trial. *J Gerontol A Biol Sci Med Sci.* Dec 12 2017;73(1):59-65.

29. Bennell KL, Nelligan R, Dobson F, et al. Effectiveness of an Internet-Delivered Exercise and Pain-Coping Skills Training Intervention for Persons With Chronic Knee Pain: A Randomized Trial. *Annals of internal medicine.* Apr 4 2017;166(7):453-462.

30. Kelly JT, Reidlinger DP, Hoffmann TC, Campbell KL. Telehealth methods to deliver dietary interventions in adults with chronic disease: a systematic review and meta-analysis. *The American journal of clinical nutrition.* Dec 2016;104(6):1693-1702.

31. Association AP. *Australian Physiotherapy Association Telehealth Guidelines Response to COVID-19*2020.

32. Kelly JT, Allman-Farinelli M, Chen J, et al. Dietitians Australia position statement on telehealth. *Nutrition & Dietetics.*n/a(n/a).

33. Lawford BJ, Bennell KL, Hinman RS. Consumer Perceptions of and Willingness to Use Remotely Delivered Service Models For Exercise Management of Knee and Hip Osteoarthritis: A Cross-Sectional Survey. *Arthritis care & research.* May 2017;69(5):667-676.

34. Lawford BJ, Delany C, Bennell KL, Hinman RS. "I was really sceptical...But it worked really well": a qualitative study of patient perceptions of telephone-delivered exercise therapy by physiotherapists for people with knee osteoarthritis. *Osteoarthritis Cartilage.* Jun 2018;26(6):741-750.

35. Lawford BJ, Bennell KL, Kasza J, Hinman RS. Physical Therapists' Perceptions of Telephone- and Internet Video-Mediated Service Models for Exercise Management of People With Osteoarthritis. *Arthritis care & research.* Mar 2018;70(3):398-408.

36. Bennell KL, Keating C, Lawford BJ, et al. Better Knee, Better Me™: effectiveness of two scalable health care interventions supporting self-management for knee osteoarthritis - protocol for a randomized controlled trial. *BMC Musculoskelet Disord.* 2020;21(1):160-160.

37. Boutron I, Altman DG, Moher D, Schulz KF, Ravaud P. CONSORT Statement for Randomized Trials of Nonpharmacologic Treatments: A 2017 Update and a CONSORT Extension for Nonpharmacologic Trial Abstracts. *Annals of internal medicine.* Jul 4 2017;167(1):40-47.

38. https://anzmusc.org.

39. Hoffmann TC, Glasziou PP, Boutron I, et al. Better reporting of interventions: template for intervention description and replication (TIDieR) checklist and guide. *BMJ.* 2014;348:g1687.

40. Chan AW, Tetzlaff JM, Altman DG, et al. SPIRIT 2013 statement: defining standard protocol items for clinical trials. *Annals of internal medicine.* Feb 5 2013;158(3):200-207.

41. Bellamy N. Osteoarthritis clinical trials: candidate variables and clinimetric properties. *Journal of Rheumatology.* 1997;24(4):768-778.

42. Nilsdotter AK, Lohmander LS, Klässbo M, Roos EM. Hip disability and osteoarthritis outcome score (HOOS)--validity and responsiveness in total hip replacement. *BMC Musculoskelet Disord.* 2003;4:10-10.

43. Osborne RH, Hawthorne G, Lew EA, Gray LC. Quality of life assessment in the community-dwelling elderly: validation of the Assessment of Quality of Life (AQoL) Instrument and comparison with the SF-36. *Journal of Clinical Epidemiology.* Feb 2003;56(2):138-147.

44. Henry JD, Crawford JR. The short-form version of the Depression Anxiety Stress Scales (DASS-21): construct validity and normative data in a large non-clinical sample. *The British journal of clinical psychology.* Jun 2005;44(Pt 2):227-239.

45. Shelby RA, Somers TJ, Keefe FJ, et al. Brief fear of movement scale for osteoarthritis. *Arthritis care & research.* 2012;64(6):862-871.

46. Ames GE, Heckman MG, Grothe KB, Clark MM. Eating self-efficacy: development of a short-form WEL. *Eating behaviors.* 2012;13(4):375-378.

47. Kellgren JH, Jeffrey MR, Ball J. *The Epidemiology of Chronic Rheumatism: Atlas of Standard Radiographs.* Vol Volume 2. Oxford: Blackwell Scientific; 1963.

48. Sangha O, Stucki G, Liang MH, Fossel AH, Katz JN. The Self-Administered Comorbidity Questionnaire: a new method to assess comorbidity for clinical and health services research. *Arthritis Rheum.* Apr 15 2003;49(2):156-163.

49. Altman R, Alarcón G, Appelrouth D, et al. The American College of Rheumatology criteria for the classification and reporting of osteoarthritis of the hip. *Arthritis Rheum.* May 1991;34(5):505-514.

50. Grima M, Dixon JB. Obesity Recommendations for management in general practice and beyond. *Australian Family Physician.* 2013;42(8):532-541.

51. Australia ESS. *Adult Pre-Exercise Screening System (APSS)*2019.

52. Borg G. *Borg's perceived exertion and pain scales*. Champaign, IL, US: Human Kinetics; 1998.

53. Sumithran P, Prendergast LA, Delbridge E, et al. Ketosis and appetite-mediating nutrients and hormones after weight loss. *Eur J Clin Nutr.* Jul 2013;67(7):759-764.

54. Bennell KL, Nelligan RK, Rini C, et al. Effects of internet-based pain coping skills training before home exercise for individuals with hip osteoarthritis (HOPE trial): a randomised controlled trial. *Pain.* Sep 2018;159(9):1833-1842.

55. McNeish D. Small Sample Methods for Multilevel Modeling: A Colloquial Elucidation of REML and the Kenward-Roger Correction. *Multivariate Behavioral Research.* 2017/09/03 2017;52(5):661-670.

Justification for Protocol version 2.0		
**Date**	**Change**	**Version no.**
6.10.2021	Dr Karen Lamb join added as a named researcher. She will support and guide Ms Fiona McManus (biostatistician) with the statistics for the ECHO study. Dr Lamb will provide oversight during the course of the analysis and reporting.	V2.0<br><br>Gained ethics approval on 6.10.2021
	Added weight management prescription medications to the Health Costs Data questionnaire at 6- and 12 months. We would like to know if participants use these medications for weight maintenance between baseline and the 6-month timepoints and between the 6- and 12-month timepoints.	
