# Supplementary material for: Effects of adding a diet intervention to exercise on hip osteoarthritis pain: protocol for the ECHO randomized controlled trial
Source: BMC Musculoskelet Disord. 2022 Mar 5;23:215. doi: 10.1186/s12891-022-05128-9 (PMC8898434; doi:10.1186/s12891-022-05128-9)
Supplement: Supplementary file 2 — Additional file 2. ECHO study participant consent form. [file 12891_2022_5128_MOESM2_ESM.pdf]

Consent Form

Centre for Health, Exercise and Sports Medicine
Department of Physiotherapy, School of Health Sciences

THE UNIVERSITY OF MELBOURNE

Title: **E**ffe**C**ts of a weight loss program in people with **H**ip **O**steoarthritis: the ECHO study

Responsible Researcher: Professor Kim Bennell
Co-investigators: Dr Michelle Hall, Prof Rana Hinman, Ms Gabby Knox, Ms Libby Spiers, Mr Sam Croft, Mr Nick Murphy

1. I consent to participate in this study, the details of which have been explained to me, and I have been provided with a written plain language statement to keep.

2. I understand that after I sign and return this consent form it will be retained by the researcher.

3. I understand what my participation will involve, and I agree that the researcher may use the results as described in the plain language statement.

4. I acknowledge that:

(a) the possible effects of participating in the program have been explained to my satisfaction;

(b) I have been informed that my participation in this study is completely voluntary and if I do not wish to take part, I am under no obligation to do so. I am free to withdraw from the project at any time without explanation or prejudice and to withdraw any unprocessed data I have provided;

(c) the study is for the purpose of research;

(d) I have been informed that the confidentiality of the information I provide will be safeguarded subject to any legal requirements;

(e) I wish to receive a copy of the summary project report of research findings at the end of the study ☐ yes ☐ no (please tick)

5. I consent to the research team contacting my general practitioner to inform him/her of my proposed involvement in this trial, and that they may need to disclose health information (i.e. diabetes, cardiac event) about me to ensure that this trial is appropriate for me
6. I consent for my general practitioner’s contact details to be disclosed to my study dietitian in the event that my they need to refer me to my GP to discuss health-related concern(s).

I, ____ consent to participate in the above study.

Participant signature Date
____
